# Regulation of sarcomagenesis by the empty spiracles homeobox genes *EMX1* and *EMX2*

**DOI:** 10.1038/s41419-021-03801-w

**Published:** 2021-05-20

**Authors:** Manuel Pedro Jimenez-García, Antonio Lucena-Cacace, Daniel Otero-Albiol, Amancio Carnero

**Affiliations:** 1 Instituto de Biomedicina de Sevilla (IBIS), Hospital Universitario Virgen del Rocío, Universidad de Sevilla, Consejo Superior de Investigaciones Científicas, Sevilla, Spain; 2 CIBER de Cancer, IS Carlos III, Madrid, Spain; 3 Department of Cell Growth and Differentiation, Center for iPS Cell Research and Application, Kyoto University, Kyoto, Japan

**Keywords:** Cancer stem cells, Sarcoma

## Abstract

The EMX (Empty Spiracles Homeobox) genes *EMX1* and *EMX2* are two homeodomain gene members of the EMX family of transcription factors involved in the regulation of various biological processes, such as cell proliferation, migration, and differentiation, during brain development and neural crest migration. They play a role in the specification of positional identity, the proliferation of neural stem cells, and the differentiation of certain neuronal cell phenotypes. In general, they act as transcription factors in early embryogenesis and neuroembryogenesis from metazoans to higher vertebrates. The *EMX1* and *EMX2*’s potential as tumor suppressor genes has been suggested in some cancers. Our work showed that *EMX1*/*EMX2* act as tumor suppressors in sarcomas by repressing the activity of stem cell regulatory genes (*OCT4*, *SOX2*, *KLF4*, *MYC*, *NANOG*, *NES*, and *PROM1*). EMX protein downregulation, therefore, induced the malignance and stemness of cells both in vitro and in vivo. In murine knockout (KO) models lacking *Emx* genes, 3MC-induced sarcomas were more aggressive and infiltrative, had a greater capacity for tumor self-renewal, and had higher stem cell gene expression and *nestin* expression than those in wild-type models. These results showing that EMX genes acted as stemness regulators were reproduced in different subtypes of sarcoma. Therefore, it is possible that the EMX genes could have a generalized behavior regulating proliferation of neural crest-derived progenitors. Together, these results indicate that the *EMX1* and *EMX2* genes negatively regulate these tumor-altering populations or cancer stem cells, acting as tumor suppressors in sarcoma.

## Introduction

Sarcomas are a type of tumor affecting a low percentage of the population and are malign and commonly highly aggressive. They are a heterogeneous group of tumors with more than 50 different histopathological subtypes according to WHO Classification of Tumours Editorial Board, 2020, giving complexity to their study^1,2^. As many sarcomas have their origin in the differentiation of neural crest-derived multipotent cells^3–6^, certain genes involved in the differentiation of this main embryological feature, such as *EMX1/2* genes, may be deregulated in sarcomas.

The neural crest is a transient embryonic structure of multipotent cells that contributes to the formation of multiple tissues^7^. Cells derived from the neural crest have developed specialized mechanisms to promote their transition from an epithelial to a mesenchymal phenotype, a process called mesenchymal–epithelial transition^8,9^. Deregulations at the level of these characteristics can become a risk by contributing to the processes of tumorigenesis and metastasis^3,4,8–11^. Cells derived from the neural crest contribute to the development of multiple cell and tissue types, such as melanocytes, Schwann cells, cells of the nervous system, adrenal medulla, and derivatives of cartilage and bones of the cephalic region, which require a highly orchestrated transcriptomic gene program. This program depends on cellular and extracellular signals that regulate migration, proliferation, differentiation, and long-distance survival to give rise to derived histotypes^8,9,12^. In general terms, sarcomas that derive from the neural crest have been proposed to be schwannoma sarcomas, malignant peripheral nerve sheath tumors, neurothekeomas, rhabdoid tumors, and synovial sarcomas^2,13–16^.

The EMX (Empty Spiracles Homeobox) genes *EMX1* and *EMX2* are two homeodomain genes homologous to the *Drosophila melanogaster* ems (empty spiracles) gene^17^. The EMX1/EMX2 proteins are members of the EMX family of transcription factors, primarily confined to the developing brain, where they play a role in the specification of positional identity, the proliferation of neural stem cells, and the differentiation of certain neuronal cell phenotypes^17^. Specifically, these genes are involved in establishing the regional pattern of the anterior brain, directly intervening in neural precursors. EMX1/EMX2 could have a role as inhibitors of cell proliferation, of the migration of cortical neuroblasts, and of the regulation of their differentiation^18–21^. In addition, Emx2 is expressed at high levels in adult neural stem cells (ANSCs) in vitro and is downregulated after differentiation. The overexpression of the *Emx2* gene in ANSCs has an antiproliferative effect but does not influence a particular differentiation pathway.

The *EMX1*/*EMX2* genes have been associated with cancer in a few solid tumors of epithelial origin, such as lung cancer^22,23^, malignant pleural mesothelioma^24^, gastric cancer^25^, endometrial cancer^26,27^, and liver metastases from colorectal adenocarcinoma^28^. In addition, these genes were associated with glioblastoma^29,30^ and melanoma^29,31^, cancer whose origin comes from melanocytes, a derivative of the neural crest.

In the tumors tested, restoration of EMX2 expression levels suppressed cell proliferation and invasive phenotypes, sensitized lung cancer cells for treatment, and inhibited the canonical Wnt pathway. In vitro EMX2 silencing promoted cell proliferation, invasive phenotypes, and activation of the canonical Wnt pathway, suggesting that EMX2 may be a tumor suppressor gene^25–30^. Overexpression of Emx2 under the Nes promoter (Nestin) suppressed cell proliferation in glioblastoma cell lines. The promoter was activated in neural stem cells, indicative of stem cell antagonism, and Emx2 expression^29,30,32–34^. However, the role of EMX1/EMX2 in the initiation and progression of human sarcoma, as a tumor model derived from the mesoderm and neural crest, has not yet been elucidated. Nor have the molecular pathways altered and dependent on EMX1/EMX2 levels been studied in depth. In the present work, we linked the formation of sarcomas in general with a possible common origin in the neural crest where EMX would maintain the migrating progenitors derived from this tissue with proliferative arrest.

## Results

### EMX represses tumorigenic properties in sarcoma

**Fig. 1:**
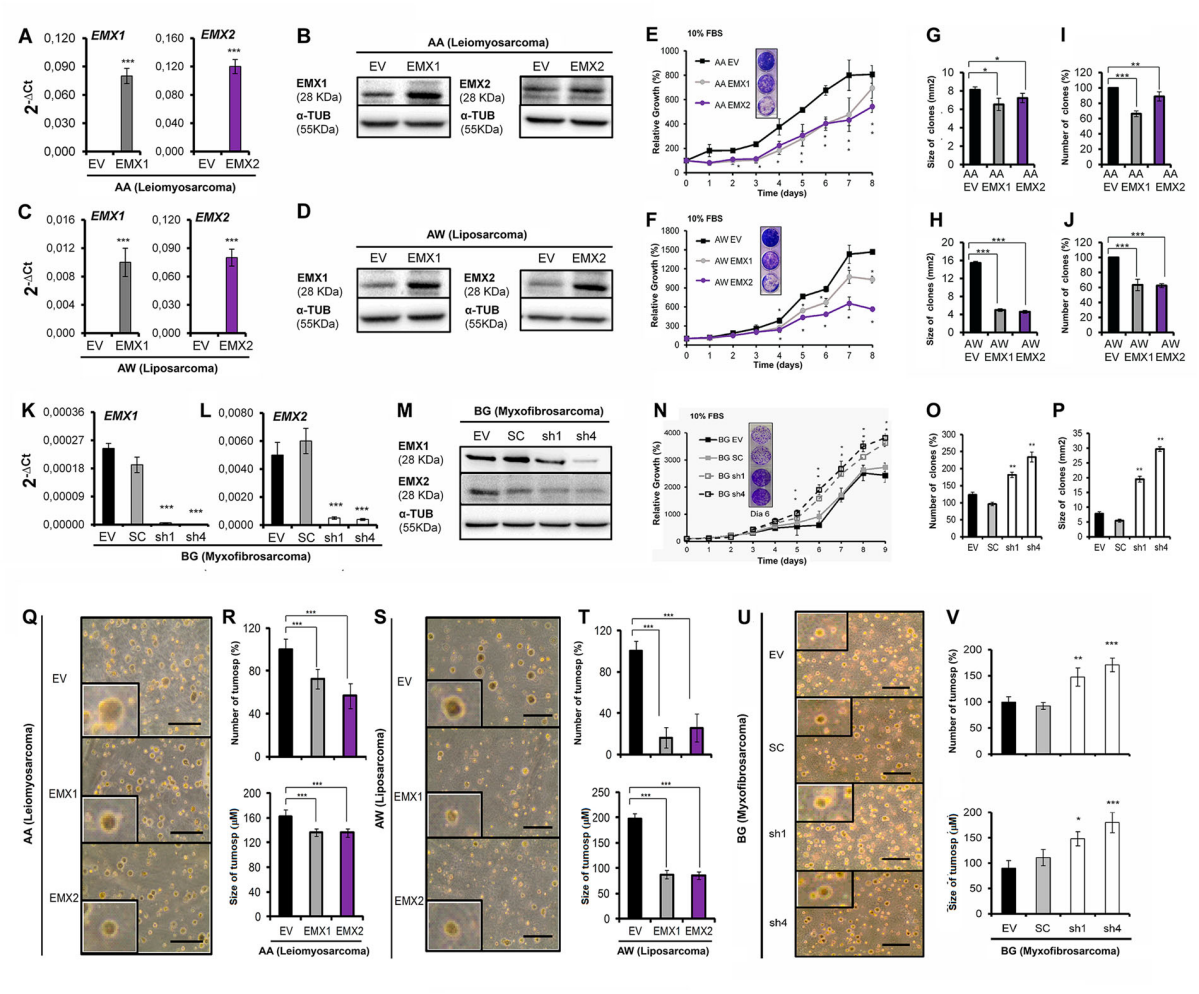
Effect of EMX1 and EMX2 expression levels in cell models expressing different levels of EMX proteins. Ectopically transfected empty vector (EV) or EMX1 or EMX2 cDNAs. In **A** and **C**, the expression levels were measured by qRT-PCR and in **B** and **D** by western blottings in the primary lines of AA sarcoma (**A**, **B**) and AW (**C**, **D**). **E**–**J** Effect of EMX1 and EMX2 overexpression on the proliferative and colony-forming capacities of sarcoma cell lines. **E**, **F** Growth curves of the overexpression of EMX1 in the AA (**E**) and AW (**F**) sarcoma lines. Inside, images of crystal violet-stained growth plate wells on the last day of the experiment. **G**–**J** The result of the clonability test assessing the effect of overexpression of EMX on the size and number of colonies in the AA (**G**, **I**) or AW (**H**, **J**) cell lines. **G**, **H** Graph of the size of the colonies. **I**, **J** Graph of the number of colonies. **K**, **L** Validation of cellular models of silencing of EMX1 and EMX2 expression levels by two independent shRNAs, sh1 and sh4, in the sarcoma line BG. **M** Protein levels of EMX1, EMX2, and the endogenous control α-tubulin (α-TUB) in the BG cell line expressing the shRNAs against EMX. **N**–**P** Effect of the reduction of EMX1 and EMX2 on the proliferative and colony-forming capacities in the sarcoma BG cell line. **N** Growth curves of the silencing models of EMX1 and EMX2 of the BG sarcoma line under the conditions of 10% FBS. Inside, images of crystal violet-stained growth plate wells on a given day of the experiment. **O**, **P** Clonability assay of the silencing models of the BG sarcoma line. **O** Graph of the number of colonies. **P** Size of the colonies. **Q**–**V** Effect of the EMX1 and EMX2 levels on anchor-independent growth. **Q**, **S**, **U** Representative images of the colonies embedded in the agar in the AA (**Q**), AW (**S**), or BG (**U**) lines with different levels of EMX protein. **R**, **T**, and **V** Percentage of the number of colonies and the mean size of the colonies for the AA (**R**), AW (**T**), or BG (**V**) cell lines with different levels of EMX protein. The horizontal bar corresponds to 500 mm. In all cases, the mean of three independent experiments is represented in triplicate ± SD. Statistical analysis was performed with Student’s *t*-test (*p < 0.05; **p < 0.01; ***p < 0.001). In western blottings, a representative experiment of at least three independently performed experiments is shown. The endogenous control was α-TUB (α-tubulin).

To explore the role of EMX, we first selected three different low-passage sarcoma cell lines from different tissues of origin; two sarcoma lines expressed low levels of EMX1 and EMX2 (Supplementary Fig. S1), as models to study the effects of EMX overexpression: AA (leiomyosarcoma) and AW (liposarcoma). These cell lines were subject to ectopic expression of EMX1 or EMX2 cDNAs, whereas the parental control was transfected with an empty vector (pCMV6-EV). All models were validated both at the mRNA level using quantitative reverse-transcriptase PCR (qRT-PCR) (Fig. 1A, C) and at the protein level (Fig. 1B, D). Very low levels of EMX mRNA detected compared to detectable proteins suggests more stable protein and rapid mRNA processing. Overexpression of the EMX genes reduced proliferation (Fig. 1E, F) and the number and size of the colonies formed (Fig. 1G–J). We also selected a third line, with high expression of EMX1 and EMX2 (BG, mixoid fibrosarcoma, Supplementary Fig. S1) as the model to reduce the levels of both genes. The reduction of the levels of EMX1/EMX2 in BG was carried out with stable expression of two different short hairpin RNAs (shRNAs) against a common sequence of EMX1 and EMX2 (pRS-sh1 and pRS-sh4). Empty vectors (pRS-EV) and vectors with random shRNA sequence (pRS-SC) were used as controls. All models were validated both at the mRNA level (Fig. 1K, L) and at the protein level (Fig. 1M). For both shRNAs, we confirmed that both EMX genes were targeted, ensuring proper downregulation of EMX signaling. In this cell line, eliminating the EMX genes increased the proliferative capacity (Fig. 1N) and the capability to form colonies (Fig. 1O, P).

**Fig. 2:**
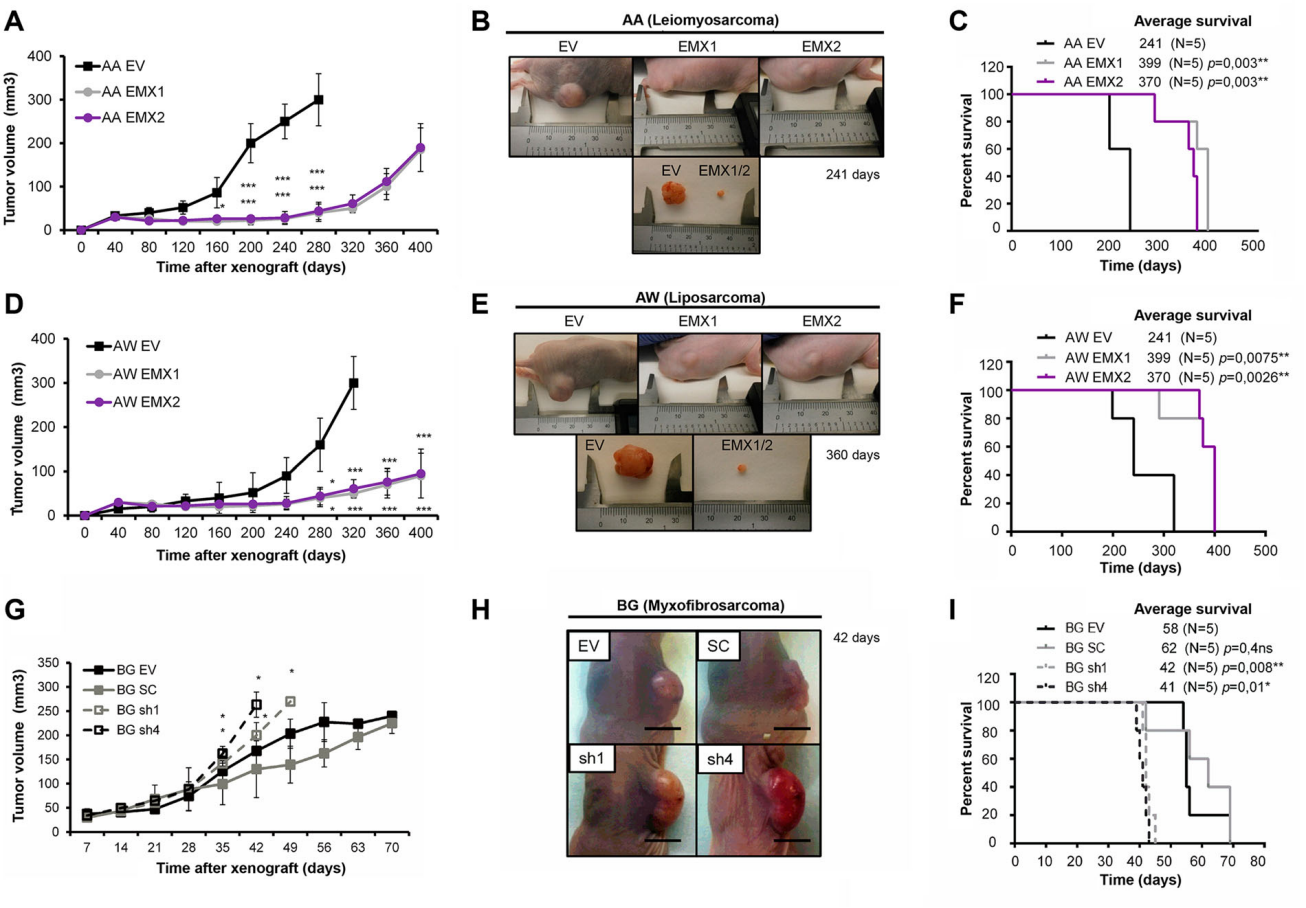
Growth in vivo of sarcoma cell lines with different levels of EMX proteins. **A**–**F** Effect of EMX1 or EMX2 overexpression on the growth of xenotransplants of AA and AW models. **A**, **D** Graph of the growth of xenograft tumors in the immunosuppressed mice with the AA (**A**) and AW (**D**) cell lines. **B**, **E** Images of the tumors in a mouse and a detailed image of the tumor size of the parental line with respect to the representative size of the lines EMX1 and EMX2 of AA (**B**) and AW (**E**). In **C** and **F**, the survival of the different cohorts of immunosuppressed mice injected with AA (**C**) and AW (**F**) is indicated. **G**–**I** Effect of reduction of EMX1 and EMX2 on tumor growth of the xenografts of the BG sarcoma cell line with different levels of EMX. **G** Tumor growth of xenograft tumors in the immunosuppressed mice from BG-derived cell lines. **H** Representative photographs of the tumors already grown in the mouse on day 42. The bar corresponds to 0.5 cm. **I** Survival of the different cohorts of immunosuppressed mice. The *p*-value of the survival graphs was obtained using the LogRank test. The rest of the analysis was performed with Student’s *t*-test (*p < 0.05; **p < 0.01; ***p < 0.001).

In all cases, we observed that high levels of EMX reduced the number and size of the colonies formed when grown in soft agar (Fig. 1Q–V). We also observed that in vivo, the xenograft tumors overexpressing EMX1 or EMX2 grew slower and were significantly smaller than the tumors formed by each of the parental lines of AA and AW (Fig. 2A, B, D, E). Furthermore, mice with xenografts from the EMX-overexpressing tumor cells survived longer (Fig. 2C, F). Similarly, with the BG line, tumors with reduced levels of EMX grew faster (Fig. 2G, H) and mice showed a marked reduction in survival compared to those with the control lines (Fig. 2I).

### The effect of the EMX1/EMX2 level on the stem cell phenotype

**Fig. 3:**
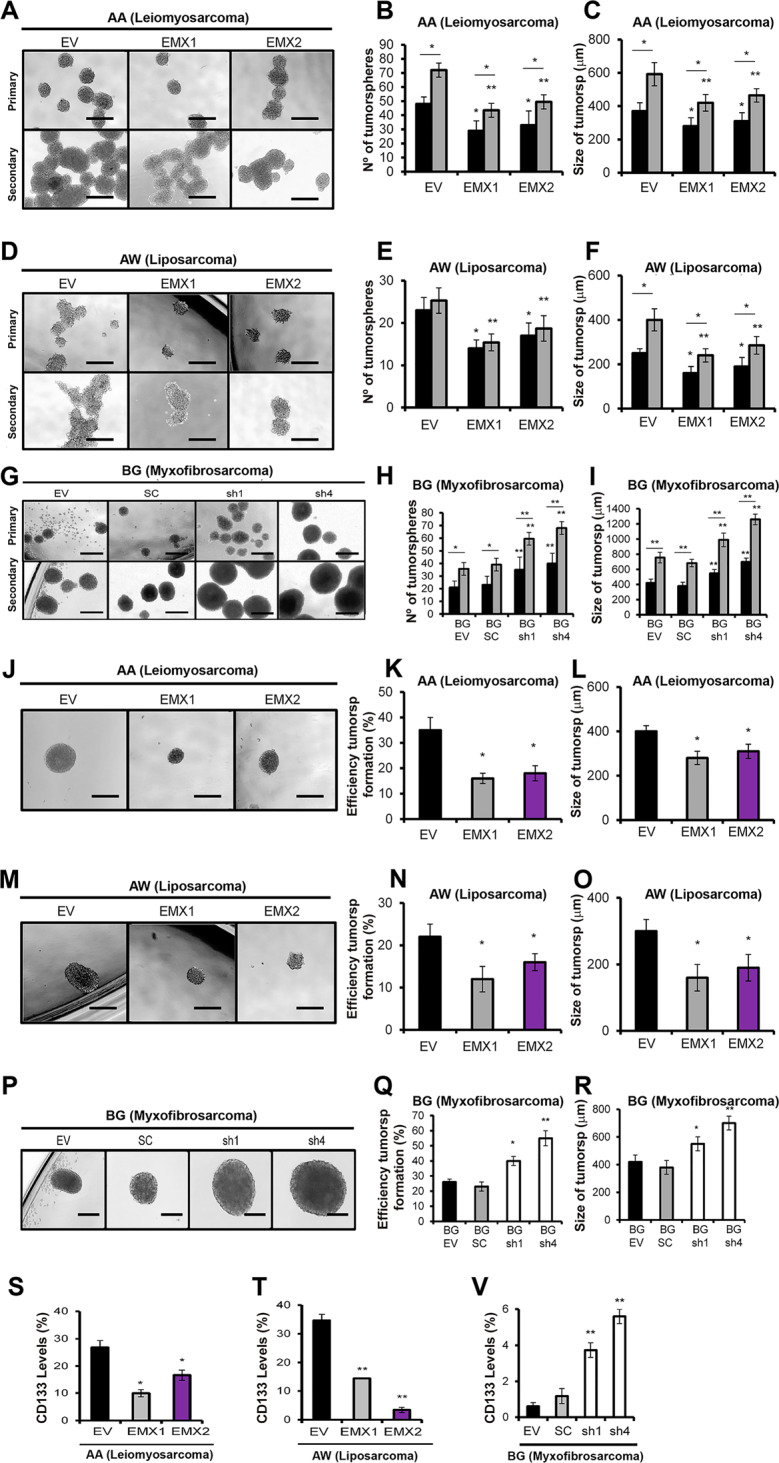
Effect of different levels of EMX1 and EMX2 on tumorigenesis in sarcoma cell lines. **A**, **D** Micrographs of the results of the primary and secondary tumor formation assays in the AA (**A**) and AW (**D**) sarcoma lines with EMX1 or EMX2 overexpression or in the BG cell line with EMXs downregulated by shRNAs (**G**). **B**, **E**, **H** Average number of primary (black bars) and secondary (gray bars) tumorspheres in AA (**B**), AW (**E**), and BG (**H**). In **C**, **F**, **I**, the mean sizes of the primary (bars in black) and secondary (bars in gray) tumors of AA (**C**), AW (**F**), and BG (**I**) are shown. **J**, **M**, **P** Representative images of tumor formation from isolated cells in the AA (**J**), AW (**M**), and BG (**P**) cell lines with different levels of EMX proteins. Sphere-formation efficiency of single cells for AA (**K**), AW (**N**), or BG (**Q**). Size of tumorspheres formed from an isolated cell in AA (**L**), AW (**O**), or BG with different levels of EMX proteins. All bars correspond to 500 µm. The mean of three independent experiments is represented in triplicate ± SD. Statistical analysis was performed with Student’s *t*-test (*p < 0.05; **p < 0.01; ***p < 0.001). **S**, **T**, **V** Effect of EMX1 and EMX2 levels on the percentage of CD133+ cells in sarcoma cell lines with different levels of EMX proteins. Graphs of the percentage of expression of the CD133 marker (conjugated with PE: CD133-PE), measured by FACS in the different models of lines AA (**S**) and AW (**T**) or BG (**V**) with different levels of EMX proteins.

Next, we performed functional tests aiming to measure the stemness properties in cells with altered EMX levels. Initially, we measured tumorspheres described as enriched in multipotent progenitors^35,36^ and have high levels of expression of tumor stem cell markers^37,38^. We measured stemness in two different ways: (1) planting 5000 cells and measuring primary and secondary tumorspheres; and (2) planting 1 single cell per well and leaving it to grow to reconstitute a cell population. Under EMX1 or EMX2 overexpression, a reduction in the number and size of primary and secondary tumorspheres was observed (Fig. 3A–F). Coincidentally, the downregulation of EMX genes promoted tumorsphere formation either in number or size (Fig. 3G–I). When the tumorsphere formation test was performed with an isolated cell by flow cytometry, the results were similar; a reduction in size and tumor formation efficiency was observed with EMX1/EMX2 overexpression (Fig. 3J–O), while the downregulation of EMX increased the efficiency and the size of the tumorspheres (Fig. 3P–R).

We analyzed the different clonal phenotypes, as depending on the phenotype the clones were classified into (i) holoclones, enriched in cells with the capacity to regenerate the culture; (ii) paraclones, formed by differentiated cells that are not capable of reconstituting a culture; and (iii) meroclones, with intermediate characteristics^39,40^. We observed a reduction of the holoclones compared to the clones with the highest degree of differentiation (meroclones and paraclones) in cells ectopically expressing EMX1/EMX2 (Supplementary Figs. FS2 and FS3).

Finally, we determined the effect on the tumor cell pool by measuring a surface marker to identify cancer stem cells (CSCs) with CD133 for sarcoma (Fig. 3S–V). In exponentially growing cells, controls with vector only or cells ectopically expressing EMX1 or EMX2 were collected, and the content in the cells positive for CD133 was measured. We observed a marked decrease in the CD133+ ratio, in the lines overexpressing the EMX genes (Fig. 3S, T). Cells with downregulated EMX genes increased the pool of CD133+ cells (Fig. 3V).

### EMX1/EMX2 repress genes related to stemness

Next, we measured the expression of some transcription factors involved in signaling pathways that regulate the core of pluripotency of stem cell factors (OCT4, SOX2, KLF4, MYC, and NANOG), as well as the expression of BMI1, NES, and PROM1, which is responsible for the CD133 antigen^41–45^.

**Fig. 4:**
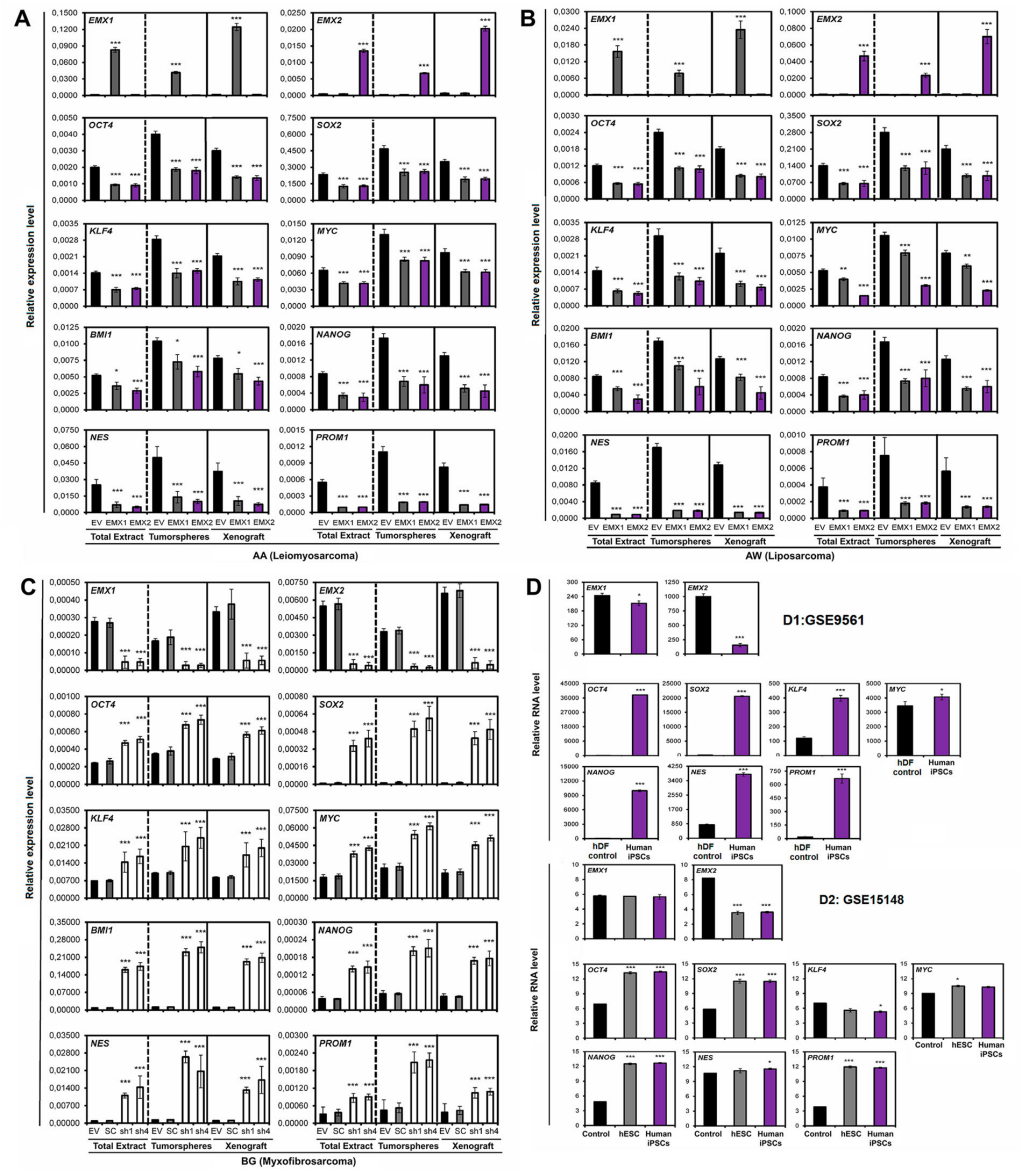
Effect of EMX1 and EMX2 levels on the expression of genes related to the phenotype of stem cells. Quantification of the relative mRNA levels of the EMX1, EMX2, OCT4, SOX2, KLF4, MYC, BMI1, NANOG, NES, and PROM1 genes by qRT-PCR in the different cell lines with different levels of EMX proteins. The graphs show the expression levels (2^−ΔCt^) of the different indicated genes for the total extract of the cell lines (ET), the tumorspheres (TO), and the xenograft tumors (XEN). **A** Line AA, **B** line AW, and **C** line BG. The average of a minimum of three independent experiments is represented in triplicate ± SD. Statistical analysis was performed with Student’s *t*-test (*p < 0.05; **p < 0.01; ***p < 0.001). **D** Transcriptional analysis of the genes *EMX1* and *EMX2*, and those related to the properties of stem cells in public databases. **D1** Analysis of the Yamanaka database (GSE9561), where the relative RNA levels of EMX1 and EMX2, and the genes related to the properties of stem cells (OCT4, SOX2, KLF4, and MYC) were measured. The database showed the transcriptome of induced pluripotent stem cells (iPSCs) generated from human dermal fibroblasts (hFDs). **D2** Analysis of the Thomson database (GSE15148), where the relative RNA levels of EMX1 and EMX2, and stem cell genes were measured in human iPSCs generated from fibroblasts of the foreskin, used as a control and in human embryonic stem cells (hESCs). Statistical analysis was performed with Student’s *t*-test (*p < 0.05; **p < 0.01; ***p < 0.001).

We measured the response of these transcripts in three different settings: whole in vitro culture (ET), cells growing in tumorspheres only (TO), or cells from the tumor growing in vivo in xenograft (XEN). We observed a significant and generalized reduction in the expression of stem cell genes under EMX1/EMX2 overexpression (Fig. 4A, B). The result presented greater differences in the expression levels in the tumorspheres (TO) with respect to the total extract of the cell line (ET) and the extract of the tumor generated in xenotransplants (XEN). In total, coincident results were obtained in the BG cell line, where reduction of EMX levels increased stemness markers (Fig. 4C).

In addition, we verified that there is a reduction in Prom1 transcription under high levels of EMX, confirming the reduction in CD133+ cells observed previously (Fig. 4A–C). A reduction in the expression of NESTIN (NES) was also observed in the EMX-positive cell population (Fig. 4A–C), reaffirming the markers NES and CD133 as markers enriched in stem cells in sarcoma and regulated by the expression of EMX. These results confirmed the negative relationship between tumor stem cell physiology and the presence of EMX1/EMX2. We observed similar correlation in bioinformatic analysis of EMX1/EMX2 in stem cell biology (Fig. 4D).

### The absence of Emx1 or Emx2 increases the tumorigenic capacity of 3MC-induced sarcomas in murine KO models

**Fig. 5:**
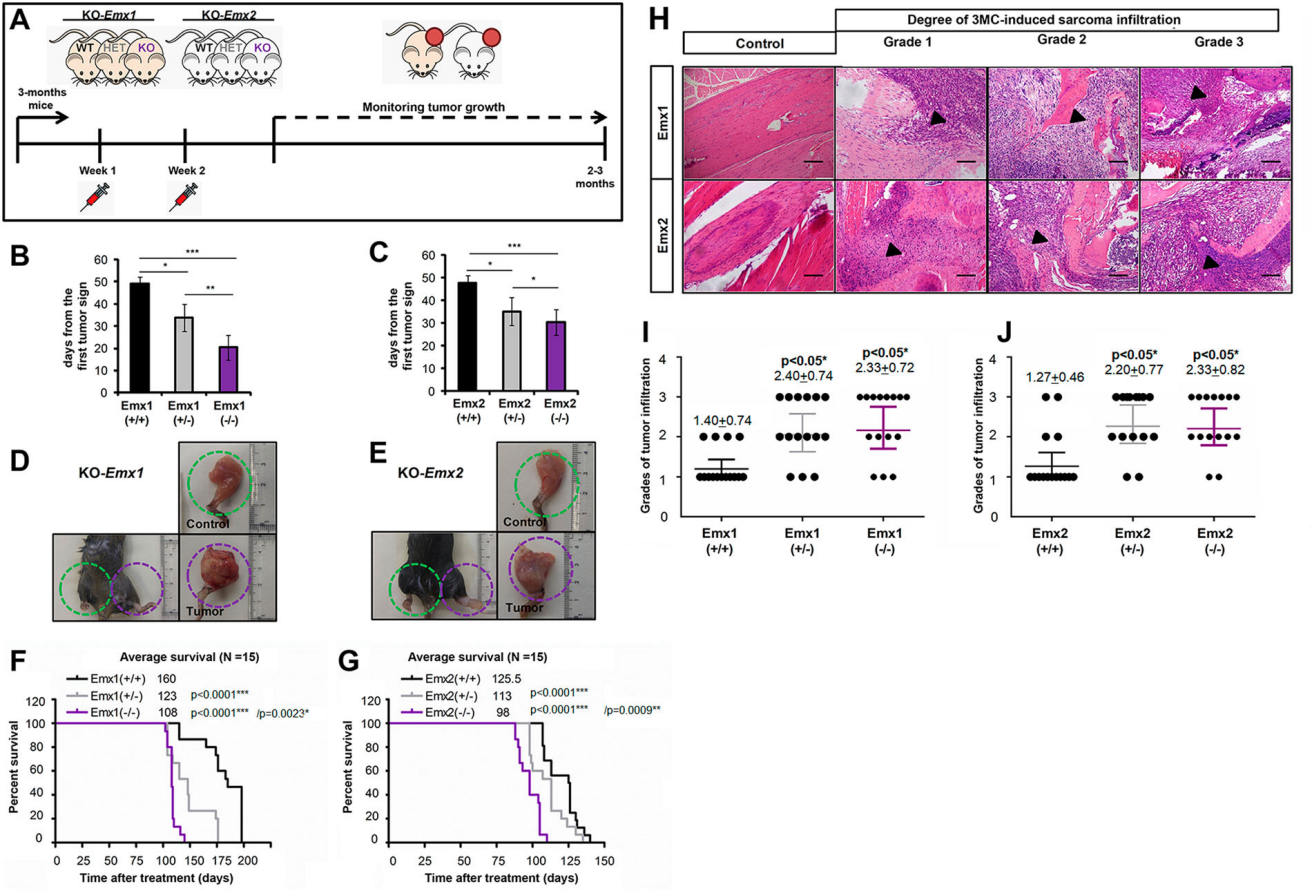
Model of sarcomagenesis in vivo induced by 3MC in the different cohorts of Emx1 or Emx2 KO mice. **A** Scheme of the experimental procedure and monitoring of intramuscular carcinogenic induction mediated by 3MC in murine models of Emx1 and Emx2. Each cohort comprised 15 mice. **B**, **C** Graph of the days since the first tumor sign in each cohort of Emx1 (**B**) and Emx2 (**C**). **F**, **G** Survival curve with respect to time post treatment with 3MC in Emx1 (**F**) and Emx2 (**G**). **D**, **E** Representative photographs of the control and tumor limbs of each Emx1 (**D**) and Emx2 (**E**) cohort. The *p*-value of the survival graphs was obtained using the LogRank test. The rest of the analysis was performed with Student’s *t*-test (*p < 0.05; **p < 0.01; ***p < 0.001). **H**–**J** Effect of Emx1 or Emx2 depletion on the tumor invasiveness of the bone marrow of the femur in the 3MC-induced sarcomas. In **H**, representative micrographs of the three grades into which the evolution of the tumor infiltration of sarcoma induced by 3MC in the bone marrow has been divided with respect to the control tissue. The points of initiation or progression of the infiltration are highlighted with the arrowhead. The bar indicates 200 µm. **I**, **J** Graphs of the degree of tumor infiltration measured in the Emx1 (**I**) and Emx2 (**J**) KO line cohorts. Each cohort had 15 mice. Statistical analysis was performed with Student’s *t*-test (*p < 0.05; **p < 0.01; ***p < 0.001).

To study the role of EMX genes in sarcomagenesis in vivo, we used a model for the specific tumor induction of sarcoma mediated by the carcinogen 3-methylcholanthrene (3MC). Thus, we established the experimental cohorts of 15 mice in both the *Emx1* and *Emx2* knockout (KO) mouse lines^46,47^ and performed the 3MC carcinogen-mediated tumorigenic induction. We used two doses of intramuscular 3MC at 1 mg in the abductor muscle of the left paw of each mouse (Fig. 5A). We followed each animal for the first tumor signs and they were killed when the tumors reached a size of 1.7 cm^3^. Then, a complete autopsy was performed, observing the state of each organ and possible metastases. Tumors were observed to appear earlier with a statistically significant difference in the Emx1 and Emx2 KO (−/−) line in a gene dose-dependent manner (Fig. 5B, C). Mice null for *Emx* developed sarcoma earlier than heterozygous mice. In addition, the data on the appearance of the tumors correlated with the significant increase in tumor size (Fig. 5D, E) and a reduction in the survival of the *Emx*-null mice (Fig. 5F, G). To verify the physiological cause of reduced survival and increased tumor malignancy, we performed a histopathological evaluation of bone infiltration of the obtained tumor sections, comparing them with control muscle tissues. After analyzing the sections of all the cohorts of both murine lines, we classified the different degrees of infiltration of the femoral bone marrow into three groups as follows: (1) incipient bone infiltration, (2) patent bone infiltration, and (3) aggressive bone infiltration (Fig. 5H). We observed a statistically significant increase in grade 3 aggressive bone infiltration in the mice with Emx downregulation (Fig. 5I, J), indicating greater malignancy and invasiveness of the tumor in the absence of the *Emx* genes.

The absence of Emx1 or Emx2 increased inflammation in the 3MC-induced sarcomas (Supplementary Fig. FS4); we observed that all induced tumors in all genotypes showed similar KI67 immunostaining levels (60–65 ± 12%) compared to 8–9 ± 2% in non-tumoral tissue sections. This finding indicates the homogeneity of tumor induction in the model mediated by the carcinogen 3MC. We also observed a greater presence of both leukocytes measured by CD45 (Supplementary Fig. FS5) and macrophages measured by F4/80 (Supplementary Fig. FS5) in the hemizygous genotypes (+/−) and homozygous null genotypes (−/−) of both murine models.

### The absence of Emx1 or Emx2 increases the phenotype of stem cells in the 3MC-induced sarcomas

To correlate the results obtained in vitro and in vivo with respect to the relationship of the properties of stem cells with the EMX genes in the primary sarcoma lines, we studied the genes regulating the properties of stem cells in the two models. For that, we (1) measured protein levels by immunohistochemistry and (2) measured mRNA levels using qRT-PCR of Nanog, Nes, Oct4, Sox2, Klf4, and Myc, both in the induced sarcoma with respect to the control muscle tissues.

**Fig. 6:**
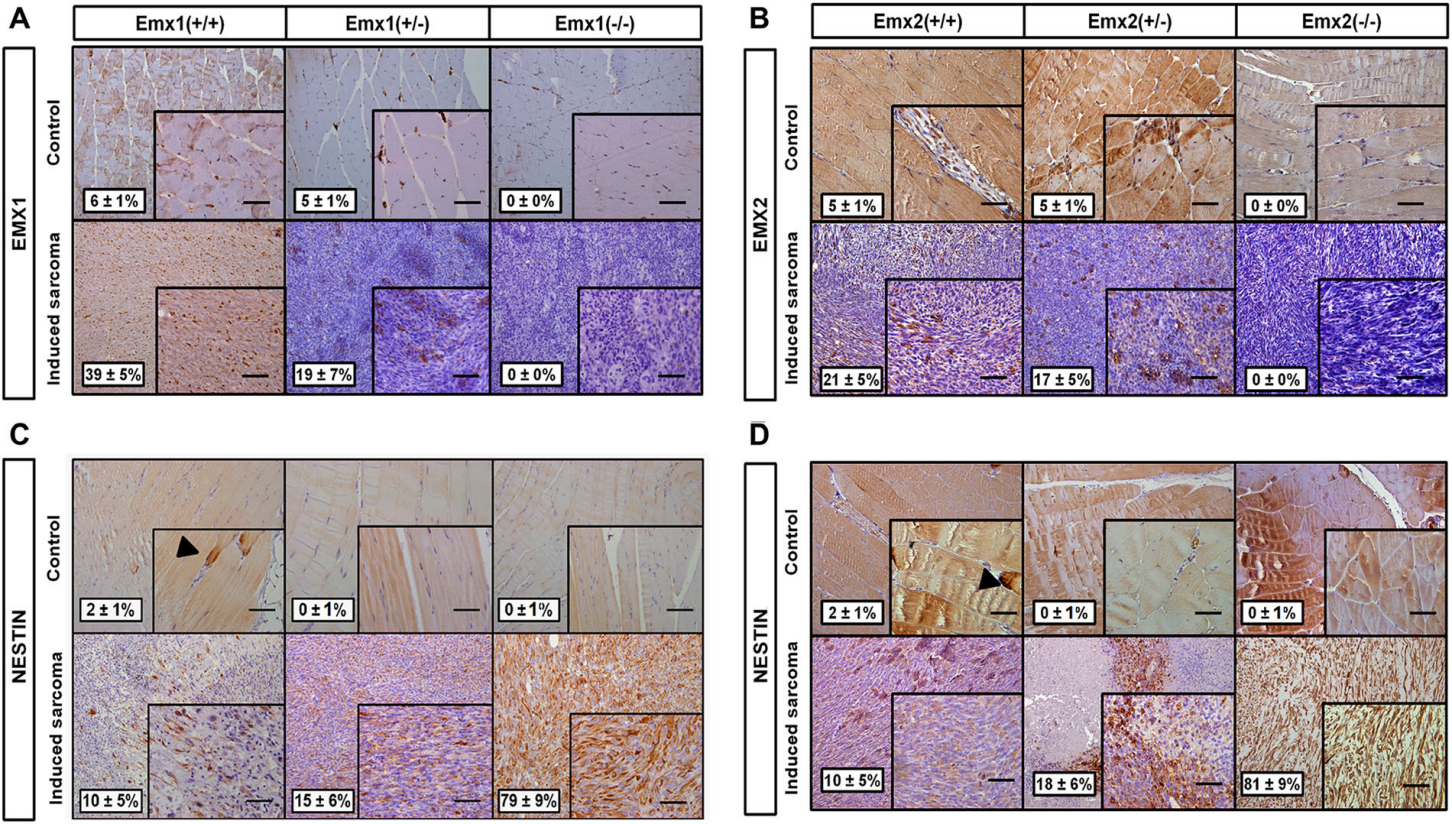
Immunophenotypic characterization of EMX1 and NESTIN in sarcomas obtained by induction of 3MC in the Emx KO mice. The results of immunohistochemistry against the transcription factor EMX1 (**A**) and EMX2 (**B**), and the neural lineage marker NESTIN in the EMX1 KO cell line model (**C**) or EMX2 KO model (**D**). The upper panel of each section shows micrographs of the control muscle tissue section at ×200 magnification and the lower panel shows micrographs of the 3MC-induced sarcoma in the Emx model. The arrow indicates a NESTIN-positive cellular immunostaining detail. The image in the lower right corner of each micrograph is ×400 magnification. The bar indicates 200 µm. The percentage of mean cell immunolabeling measured in five fields per replicate ± the SD was monitored. Immunohistochemistry was performed in a minimum of three replicates for each tumor sample.

We observed 5–6 ± 1% *Emx*-positive nuclei in the control tissue and 21–39% ± 5% in the induced sarcoma tissue (Fig. 6A, B), indicating an increase in cells with expression of EMX in sarcoma, with no expression observed in the Emx-null compared to the wild genotype (Fig. 6A, B). This result negatively correlates with the protein expression of the neural marker NES, which has its highest level of expression in sarcoma tissues of the *Emx*-null mice. In contrast, NES presented very low levels in the induced sarcoma tissues of the wild-type (WT) mice, low levels in the WT genotype, and no levels in the control tissues of the hemizygous and recessive homozygote genotypes (Fig. 6C, D). These results together suggest a possible origin on neural crest-derived progenitors of these induced sarcomas in the EMX-downregulated mice.

**Fig. 7:**
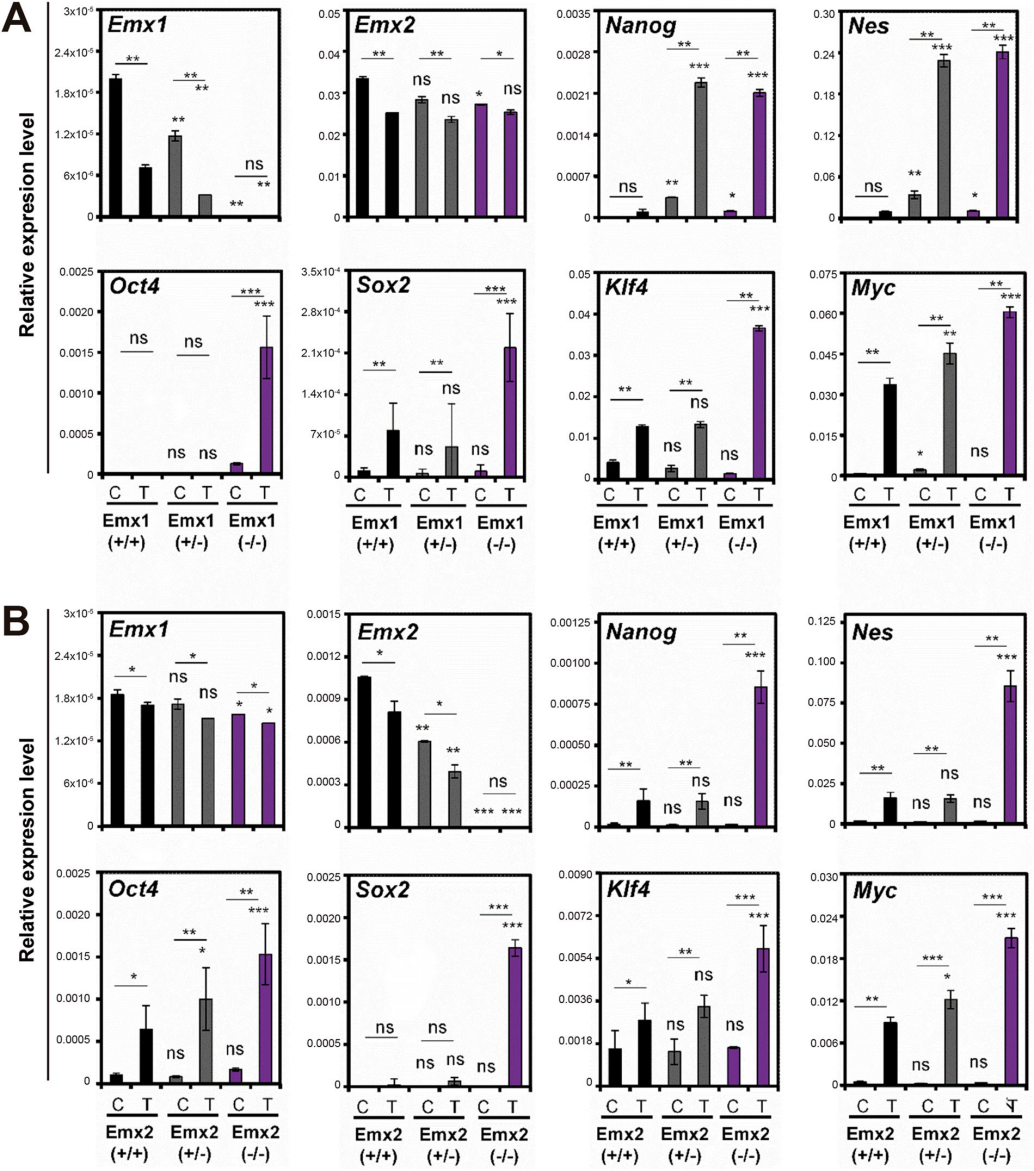
Analysis of the expression levels of the regulatory genes of stem cell biology in 3MC-induced sarcomas of the Emx1 and Emx2 KO mice. **A**, **B** Results of the relative expression (2^−ΔCt^) of Emx1, Emx2, Nanog, Nes, Oct4, Sox2, Klf4, and Myc in the different murine cohorts of Emx1 (**A**) and Emx2 (**B**) KO mice. The control muscle tissue is C and 3MC-induced sarcoma tumor tissue (T). The mean of a minimum of three independent experiments is represented in triplicate ± SD. Statistical analysis was performed with Student’s *t*-test (*p < 0.05; **p < 0.01; ***p < 0.001).

Finally, a molecular characterization of the genes of the stem cells was performed, and the *Emx1* and *Emx2* genes were measured by qRT-PCR to characterize the two murine KO models, as well as the *Nanog*, *Nes*, *Oct4*, *Sox2*, *Klf4*, and *Myc* genes (Fig. 7). In the murine Emx1 model, a reduction in Emx1 levels was obtained in the heterozygous Emx(+/−) mice and no expression was found in the Emx(−/−)-null homozygotes (Fig. 7), with lower expression levels in induced sarcoma (T) than in control muscle tissue (C). We observed an increase in the *Nanog*, *Nes*, *Oct4*, *Sox2*, *Klf4*, and *Myc* mRNA levels in the mice with a functional allele of *Emx*(+/−) and especially in the null Emx(−/−), with higher levels in the induced sarcoma compared to the control muscle tissue (Fig. 7).

These results indicate that the reduction of Emx1/Emx2 allows the expression of genes related to the properties of stem cells (*Nanog*, *Nes*, *Oct4*, *Sox2*, *Klf4*, and *Myc*), confirming this overexpression in the case of induced sarcomas and in the null murine models of both genes. This result indicates that *Emx* genes are negatively correlated with genes related to stem cell properties in vivo. Analysis of overall survival in sarcoma databases with respect to EMX1 and EMX2 levels suggest that, in general terms, a reduction in disease-free survival or/and overall survival was observed in the group of patients with low levels of EMX1 and EMX2 (Supplementary Fig. FS6).

## Discussion

The transcription factors EMX1 and EMX2 act as tumor suppressors in sarcomas in vitro and in vivo by decreasing the expression of genes regulating the properties of stem cells and the stem cell phenotype. The tumor suppressor function of EMX found in this work is consistent with those previously obtained from the overexpression of EMX2 in lung cancers^22,48,49^, colorectal metastasis^28^, gastric cancer^25^, endometrial cancer^26,27^, and glioblastoma^29,30^. We found that the overexpression of EMX1 or EMX2 arrests CSCs, explaining the reduction in the regeneration of the culture or in the number and size of tumorspheres and tumorigenicity in vivo. This reduction in the CSC population dependent on EMX levels may be linked to reports associating a better prognosis of these tumors with EMX2 overexpression. The results indicated a reduction in the number, size, and efficiency of tumor formation, as well as a negative correlation of the CD133+ fraction with the EMX1 and EMX2 levels.

In addition, the negative correlation of stem cell genes (*OCT4*, *SOX2*, *KLF4*, *MYC*, *NANOG*, *NES*, and PROM1) with EMX1/EMX2 was verified in culture in vivo and in the in silico transcriptional analysis of the databases of embryonic and human induced pluripotent progenitors. Taken together, these results indicate that the EMX genes retain their ontogenetic function of blocking stemness. In the neural embryonic context, cell proliferation is reduced by directly blocking Sox2^50,51^ and other precursors (such as Sox3 and Sox11) related to neural development. This process allows the creation of a gene expression profile that regulates the differentiation and migration of neural precursor cells in neurospheres cultured in vitro^40^.

Despite the different molecular processes by which sarcomagenesis is reported to occur, EMX1 and EMX2 negatively contribute to sarcomagenesis in vivo by regulating stemness. The 3MC carcinogen-induced sarcomas have a more aggressive phenotype, increased infiltration into the femoral bone marrow, and reduced survival in the Emx1/Emx2 KO mice. This increased malignancy may be due to increased expression of stem cell genes in both the precursors of Emx1/Emx2 KO mice, as mature tissue does not have EMX expression (Fig. 6). In addition, the Emx1/Emx2 KO mice that are induced with sarcoma have decreased survival. These results can be explained if the induced sarcoma occurs as a consequence of the tumor transformation of the stem cells, which, in the absence of EMX proteins, facilitates the process of tumor transformation. One of the most representative neural proteins of stem cell regulation is NES^52,53^. A marked increase in NES was obtained in the tumors of both KO mice. These data correlate with our data for EMX1/EMX2 depletion found in the CD133+ fraction of human sarcoma primary lines. In other works, the presence of NES was associated with increased tumor malignancy and tumor stem cell populations in the tumor^53^.

Comparing the control muscle tissues and the induced sarcoma, we observed the highest expression of the stem cell genes (*Oct4*, *Sox2*, *Klf4*, *Myc*, and *Nanog*) and aggresivity in the sarcomas induced in the Emx1 or Emx2 KO mice. These results indicate that sarcomagenesis occurs mainly in those populations or cell progenitors of the muscle tissue of KO animals that overexpress stem cell genes, such as NES. This overexpression of pluripotency genes in combination with the 3MC carcinogen enables sarcomas to develop and tumor malignancy to increase, as demonstrated in other studies^54–58^. Therefore, all these data explain the negative correlation between the EMX genes and the properties of CSCs.

In embryonic studies of Emx1 and Emx2 in the development of neural progenitors in mice, expression was found in the dorsal region of the neural tube, allowing us to constitute an expression gradient that affects not only the neural tube but also the neural crest by direct proximity of the gradient^40,50,51,59–61^. Thus, as the process of ontogenetic development in the neural tube and neural crest progresses with the successive stages of cell differentiation, the levels of Emx expression gradually decrease, leading to the differentiation of the different tissue subtypes^51,60^. Therefore, in the progressive reduction of the expression of the EMX genes, it is expected that the expression of the stem cell genes will be allowed, as well as proliferative regulators. Thus, at this level of differentiation with loss of EMX, other signaling pathways or genes could participate in ontogenetic processes. EMX genes are essential in the early stages of development by inducing cell differentiation of different neural precursors.

The *EMX1*/*EMX2* genes are expressed in neural precursors (neuro-ectodermal), inducing cell differentiation, proliferation, and migration. After the development processes in which the EMX genes are believed to induce cell differentiation in early precursors, their hypermethylation and silencing in mature cells take place but are maintained in stem cells regulating their stem proliferative arrest. EMX lost by methylation in stem cells may be considered the first “hit” that allows the activation of certain genes in stem cells and further carcinogenic insult will promote tumorigenesis.

All together, our results suggest that sarcoma self-renewing populations originate from neural or mesodermal precursors where *EMX1* and/or *EMX2* expression is silenced. The processes of cellular differentiation during ontogenetic development produce the silencing of *EMX* genes by hypermethylation of their promoters in differentiating, arrested cells, whereas in individual stem cells from neural crest-derived progenitors, EMX levels are maintained at high levels, controlling proliferation.

We propose that the tumor suppressive effect of EMX migth be extrapolated to the heterogeneity of sarcomas, although experiments in many other different sarcoma types should be performed to corroborate this hypothesis. This is because their behavior as tumor suppressors is robust in several different primary sarcoma lines (leiomyosarcoma, liposarcoma, and myxoid fibrosarcoma), xenografts thereof, a murine model of sarcomagenesis in vivo, and sarcoma tumors from the databases of analyzed patients. Sarcomas are a very heterogeneous group and it has been reported that they do not have a single embryological origin; most seem to derive from mesodermal precursors^1,62–64^ and some from precursors of the neural crest^3–5,65^. Furthermore, head and neck sarcomas that derive exclusively from cartilage, bone, muscle, and adipose tissue have been proposed to originate from the neural crest^4^. Furthermore, we proposed that EMX1/EMX2 transcription factors could block the expression of essential stem cell genes during sarcomagenesis.

These findings may suggest why the cell population of neural-like and mesenchymal-like precursors with the enhanced multipotent properties are those with null expression of EMX1/EMX2 and high levels of NES. Our model adapts to the existing developmental model, adding the data that certain populations of CSCs positive for the analyzed stem cell markers and dependent on EMX are the ones that can give rise to many sarcomas, regardless of the embryological origin, of the histology of the tumor, and the place of appearance. However, much more research is needed to confirm this point.

Until now, we have considered *EMX1* and *EMX2* as two genes with the same function based on the results obtained in our work. It should be noted that the scientific literature in general only mentions EMX2, either ontogenetically or oncologically. However, it is expected that EMX1 and EMX2 could have different functions, although they may be partially or completely redundant during sarcomagenesis.

In summary, our work showed that EMX1 and EMX2 act as tumor suppressors by suppressing the activity of stem cell regulatory genes (*OCT4*, *KLF4*, *MYC*, *SOX2*, *NANOG*, *NES*, and *PROM1*) in sarcoma. It is proposed that there is no or low expression of EMX1 and EMX2 in CSCs embedded in the tissue of origin of the sarcoma, allowing self-renewal of the tumor. Following overexpression of EMX transcription factors, there is a reduction in tumorigenesis and the capacities for self-renewal and maintenance of CSCs in primary sarcoma lines and in xenografts thereof in vivo. Murine KO models of Emx1 or Emx2 have been used to validate the initiation and development of sarcomagenesis in sarcomas induced with wild or hemizygous levels of Emx1/Emx2. The induced sarcoma of the KO group is more aggressive, more infiltrative, and has a greater capacity for tumor self-renewal than that of the WT group. Based on this evidence, EMX genes are proposed as possible new predictive biomarkers of response in sarcoma, where they play a fundamental role as tumor suppressor genes regulating various cellular genes.

## Materials and methods

### Transfections and plasmids

Subconfluent cells were transfected with TransIT-X2 reagent (Mirus) according to the manufacturer’s instructions. At 48 h, the cells were seeded in 10 cm plates with media containing the appropriate selection drug (100–450 μg/ml G418, 0.25–0.4 μg/ml puromycin, or 0.25–0.4 μg/ml blasticidine). The cells were transfected with the plasmids from Table 1.

**Table 1:** Plasmids used in this work.

Plasmid	Insert sequence	R. eukar.	Origin
pRS-SC sh	-	Puro	Origene (TR313216)
pRS-sh1-EMX1	5′-GCTTCAATTTAAGCCACAGTGTCTCCGAG-3′	Puro	Origene (TI363329)
pRS-sh4-EMX1	5′-GGCAGTCTCAGCCTCTCCGAGACGCAGGT-3′	Puro	Origene (TI363332)
pRS-sh1-EMX2+EMX1	5′-TCAAGCCATTTACCAGGCTTCGGAGGAAG-3′	Puro	Origene (TI352857)
pRS-sh4-EMX2+EMX1	5′-CGGTGGAGAATCGCCACCAAGCAGGCGAG-3′	Puro	Origene (TI352860)
pCMV6-EV	-	G418	Origene (PS100001)
pCMV6-EMX1	*EMX1* ADNc	G418	Origene (RC208006)
pCMV6-EMX2	*EMX2* ADNc	G418	Origene (RC222758)

*A.* resistance, *Amp* ampicillin, *Blast* blasticidin, *EV* empty vector, *G418* geneticin, *Kan* kanamycin, *LAB* generated in the laboratory, *pRS* pRetroSuer, *Pure* puromycin, *SC* random sequence shDNA (scrambled shDNA), *T* transfection.

### Cell culture

The cell lines were obtained from the European Collection of Authenticated Cell Cultures (ECACC) commercial repository or generated in the laboratory as previously reported^66–68^. No further authentication was conducted by the authors. The cells were negative for mycoplasma. The cell lines were maintained in Dulbecco’s modified Eagle’s medium, F10, or RPMI (AQmedia; Sigma) as indicated in Table 2, supplemented with 10% fetal bovine serum (FBS) (Gibco), penicillin, streptomycin, and fungizone (Sigma).

**Table 2:** The cell lines used in this study were obtained from the ECACC commercial repository or generated in the laboratory as previously reported^67–69^.

Cell line	Species	Tissue	Culture media	Growth	Origin
A-673	Human	Ewing’s sarcoma	RPMI	Adherent	ATCC
SAOS-2	Human	Osteosarcoma	DMEM	Adherent	ATCC
SW-872	Human	Liposarcoma	DMEM	Adherent	ATCC
IMR-90	Human	Fibroblast*	DMEM	Adherent	ATCC
WI-38	Human	Fibroblast*	DMEM	Adherent	ATCC
CNIO-AA	Human	Leiomyosarcoma	F10	Adherent	LAB
CNIO-AW	Human	Liposarcoma	F10	Adherent	LAB
CNIO-AX	Human	Liposarcoma	F10	Adherent	LAB
CNIO-AZ	Human	Fibrous sarcoma	F10	Adherent	LAB
CNIO-BC	Human	MPNST	F10	Adherent	LAB
CNIO-BD	Human	Ewing’s sarcoma	F10	Adherent	LAB
CNIO-BG	Human	Mixoid fibrosarcoma	F10	Adherent	LAB
CNIO-BO	Human	Mixoid fibrosarcoma	F10	Adherent	LAB
CNIO-BP	Human	Osteosarcoma	F10	Adherent	LAB
CNIO-CE	Human	Rabdomyosarcoma	F10	Adherent	LAB
CNIO-DA	Human	Mixoid fibrosarcoma	F10	Adherent	LAB
CNIO-DD	Human	Mixoid fibrosarcoma	F10	Adherent	LAB

*Primary fibroblast.

### Transfection using the commercial TransIt-X2 kit (Mirus)

Cells were seeded in six-well plates the day before, so that they were at 80% confluence at the time of transfection. The procedure consists of mixing 2.5 µg of plasmid DNA and 250 µl of medium (amounts to transfect cells in one well). Then, 7.5 µl of TransIT-X2, a non-liposomal polymer that coats the DNA favoring its transport into the cell, is added, mixed with the pipette, and incubated for 30 min at room temperature. Subsequently, the mixture is added dropwise on the cells, which were incubated for 48 h at 37 °C to then start the selection process.

After 24–48 h of incubation after transfection or infection, the cells were trypsinized, seeded at low density from a 3 cm plate to a 10 cm plate in the case of transfected cells, and the selection with the required antibiotic was started. The selection was maintained for the time necessary for the untransfected cells to die and subcultures were obtained on the plates of the transfected cells. Once the selection was completed, for maintenance the concentration of the antibiotic was reduced by half.

#### For either EMX1 or EMX2 overexpression models with pCMV6 plasmids:

AA cell line: 1 mg/ml G418 (half concentrated for maintenance: 0.5 mg/ml);

AW cell line: 1 mg/ml G418 (half concentrated for maintenance: 0.5 mg/ml);

Ham’s F10 medium supplemented with 10% FBS and 1 mg/ml G418 for selection, and after selection 0.5 mg/ml for maintaining stable cell line.

Selection time: ~10–15 days (refreshing the culture medium every 2 days). Taking into account that EMX1 and EMX2 behave as tumor suppressors, clones that overexpress either EMX1 or EMX2 can be progressively lost, so that quantitative PCR (qPCR) measurements are periodically (once every 1–2 weeks) performed to see expression levels, otherwise the transfection was repeated.

#### For knockdown BG model with pRS plasmids:

BG cell line: 1 μg/ml G418 (half concentrated for maintenance: 0.5 μg /ml);

Ham’s F10 medium supplemented with 10% FBS and 1 μg/ml puromycin for selection for maintaining stable cell line.

Selection time: ~8–12 days (refreshing the culture medium every 2 days). Taking into account that knocking down EMX levels promotes a proliferative phenotype, this model tends not to be lost; in any case, qPCR measurements are periodically performed (once every 1–2 weeks) to see expression levels, otherwise the transfection was repeated.

In all cases 1/2 dosis of the antibiotic for selection was used for continuous culture (maintenance), to ensure the maintenance of the transfection.

### Tumorigenesis assays

Proliferation assays, clonability assays, and growth assays in soft agar, and cell migration and invasion assays were performed as indicated in refs. ^69,70^.

### Tumorsphere assay

A total of 5000 cells were seeded in triplicate in 24-well Ultra-Low Attachment Plates (Costar) containing 1 ml of MammoCult basal medium (Stem Cell Technologies) supplied with 10% MammoCult proliferation supplement, 4 μg/ml heparin, 0.48 μg/ml hydrocortisone, penicillin, and streptomycin. After 5–10 days, depending on the cell line (AA in 5–7 days, AW in 6–10 days, BG in 7–8 days), the number of primary tumorspheres formed was measured using an inverted microscope (Olympus IX-71).

Subsequently, the secondary tumorspheres were generated by collecting the medium with the tumorspheres, centrifuging for 5 min at 900 r.p.m., trypsinizing for 5 min, and centrifuging again at 900 r.p.m. for 5 min. Finally, all the cells trypsinized were resuspended in fresh, complete, supplemented MammoCult medium and 1 ml/well was seeded in a 24-well, low-stick plate.They were incubated for more 4–5 days and the number and size of the tumorspheres were measured again and quantified.

### Single-cell tumorsphere assay

Single cells were individually seeded through cell sorting with a FACS Jazz flow cytometer (BD Biosciences) in 96-well Ultra-Low Attachment Plates containing 1 ml of MammoCult basal medium (Stem Cell Technologies) supplied with 10% MammoCult proliferation supplement, 4 μg/ml of heparin, 0.48 μg/ml of hydrocortisone, penicillin, and streptomycin. After 30 days, the number of individual primary tumorspheres formed was measured using an inverted microscope (Olympus IX-71).

### Analysis and classification of clonal phenotypes

The cells were seeded at low density, 100 or 1000 depending on the cell line, in triplicate in 10 cm plates and cultured under standard conditions. The medium was changed twice a week. After 10–15 days, once the clones were formed, the morphology of each clone was observed under an inverted microscope (Olympus CKX41). Finally, each type of clone was counted based on the following classification: holoclone (more compact and rounded clone with a higher percentage of CSCs), meroclone (clone with a more irregular cell arrangement at the edges and a lower proportion of CSCs), or paraclone (greater separation of cells from each other, due to a greater degree of differentiation and lower percentage of CSCs)^71,72^.

### Marker analysis by flow cytometry

The previously trypsinized suspension of 1 × 10e6 cells was resuspended in 125 µl of phosphate-buffered saline (PBS) with 2% FBS and 5 mM EDTA. This suspension was then blocked by adding 12.5 µl of blocking agent (Miltenyi Biotec) and incubated for 10 min on ice. The anti-CD133 antibody conjugated to fluorochrome phycoerythrin (MACS) was then added at a 1 : 25 dilution and incubated for 30 min in ice and darkness. A tube with 1 × 10e6 cells was left unlabeled with the antibody to use as a negative control for staining. After the end of the incubation period, the cells were washed two times with PBS, with 2% FBS and 5 mM EDTA. Subsequently, the cells were centrifuged for 5 min at a speed of 1000 r.p.m. and resuspended in 500 µl of PBS with 2% FBS and 5 mM EDTA. Finally, the cell suspension was examined in a Canto II analytical cytometer (BD Biosciences), separating in certain cases two populations positive and negative for the CD133 marker.

### Reverse-transcriptase quantitative PCR

Total RNA from cell lines was extracted and purified using the miRNeasy kit (Qiagen) and reverse transcription was performed with 3 µg of mRNA using the High Capacity cDNA Reverse Transcription kit (Life Technologies) according to the manufacturer’s instructions. We used the following probes (Table 3):

**Table 3:** Probes for Q-PCR used in this study.

HumanGene	Probe (human)	Mouse gene	Probe (mouse)
*GAPDH*	Hs03929097_g1	*Gadph*	Mm99999915_g1
*EMX1*	Hs00417957_m1	*Emx1*	Mm01182609_m1
*EMX2*	Hs00244574_m1	*Emx2*	Mm00550241_m1
*CD133*	Hs01009257_m1		
*NES*	Hs04187831_g1	*Nes*	Mm00450205_m1
*NANOG*	Hs04260366_g1	*Nanog*	Mm02019550_s1
*OCT4*	Hs00999632_g1	*Oct4*	Mm03053917_g1
*SOX2*	Hs01053049_s1	*Sox2*	Mm03053810_s1
*KLF4*	Hs00358836_m1	*Klf4*	Mm00516104_m1
MYC*	Hs00153408_m1	*c-Myc*	Mm00487804_m1
*BMI1*	Hs00995536_m1		

*Primary fibroblast.

### Protein isolation and western blot analysis

Western blottings were performed as previously described elsewhere^69–72^. Membranes were incubated with the following primary antibodies (Table 4):

**Table 4:** Antibodies used in this study.

Antibody	Company		Dilution		
			WB	IHQ	
				DIL	DA
EMX1 (rabbit polyclonal)	Abcam	ab136102	1:1000	1:100	EDTA
EMX2 (mouse polyclonal)	Abcam	ab171818	1:1000	-	-
EMX2 (rabbit polyclonal)	Invitrogen	PA5-34415	-	1:500	TC
α-tubulin (mouse monoclonal)	Sigma-Merck	T9026	1:10,000	-	-
KI67 (rabbit monoclonal)	MAD	000310-QD	-	1:250	TC
NESTIN (rat polyclonal)	SCB	SC-101541	-	1:100	TC
Rabbit anti-mouse HRP (polyclonal)^a^	Abcam	ab97046	1:5000	-	-
Goat anti-rabbit HRP (polyclonal)^a^	Abcam	ab97051	1:5000	-	-
Goat anti-rabbit HRP (polyclonal)^a^	JAC	111-035-003	-	1:400	-
Goat anti-rat HRP (polyclonal)^a^	JAC	112-035-003	-	1:400	-
Rabbit anti-goat HRP (polyclonal)^a^	Abcam	ab97100	-	1:400	-

*CST* Cell Signaling Technologies, *DA* antigen unmasking, *DIL* dilution, *EDTA* buffer EDTA, *IHQ* immunohistochemistry, *JAC* Jackson Immuno Research, *MAD* Master Diagnostica, *TC* citrate buffer, *SCB* Santa Cruz Biotechnology, *WB* western blotting.

^a^Secondary antibodies.

The proteins were detected using an ECL detection system (Amersham Biosciences) and a Bio-Rad Chemidoc Touch.Bio-Rad’s Image Lab 5.1 program was used to quantify the protein bands. Each band was selected individually and the value was reported as a loading control on the same membrane, which was usually immunodetection for α-tubulin.

### Xenografts in nude mice

Tumorigenicity was assayed by the subcutaneous injection of 4 × 10^6^ cells into the right flanks of 4-week-old female athymic nude mice. Cells were suspended in 50 µl of Matrigel (Corning) prior to the injection. Animals were examined weekly and after 150–180 days, depending on the cell lines, the mice were killed and the tumors were extracted and conserved at −80 °C. Tumorsphere tumorigenicity was measured by seeding 10^4^ cells as described in the tumorsphere assay section. After 5 days, tumorspheres were disaggregated with trypsin, resuspended in 50 µl of Matrigel, and injected into the right flanks of 4-week-old female athymic nude mice. Animals were examined weekly and after 85–100 days, depending on the cell lines, mice were killed and tumors were extracted and conserved at −80 °C. Tumor volume (mm^3^) was measured using calipers. All animal experiments were performed according to the experimental protocol approved by the IBIS and HUVR Institutional Animal Care and Use Committee (0309-N-15).

### Mouse EMX KO lines used and maintenance of the experimental colonies

The KO mice were provided by the Japanese Natural Science Research Center RIKEN (http://www.riken.jp/en/). They were generated from TT2 embryonic stem cells derived from an F1 embryo mixed between C57BL/6J and CBA/J. The mice of the KO-Emx1 line were kept in a mixed C57BL/6J × CBA/J genetic background and the KO-Emx2 line in a pure C57BL/6J genetic background. The KO lines for Emx1 and Emx2 were maintained and crossed in heterozygosity to obtain the different genotypes of Emx1 or Emx2 in the same litter (Emx1/Emx2 (+/+), Emx1/Emx2 (+/−), and Emx1/Emx2 (−/−)), and thus reduce possible genetic variability. All mice were kept according to the standards established in the IBiS animal facility (based on the provisions of Royal Decree 53/2013) and were killed by CO_2_ inhalation when any significant sign of disease was detected to avoid the suffering of the animal.

### Carcinogenic tests

Treatment with 3MC was carried out to induce sarcomas in the abductor muscle of the murine model. For this, a solution of the carcinogen 3MC (Sigma-Merck) in sesame oil (Sigma-Merck) was prepared at a concentration of 10 mg/ml. Three-month-old mice were treated, inoculating a weekly dose of 1 mg of 3MC (100 µl), performing two doses intramuscularly in the abductor muscle of the right hind leg. The mice were examined once a week and were killed when the tumor reached a size of 1.7 cm^3^ or showed signs of disease.

### Necropsy

After the killing of each animal, a complete necropsy of each animal was performed and samples of the tumor and affected tissues were collected for histological and molecular analysis. The tumor samples were divided into three parts: one for histology and the others for RNA or protein analysis. Histology samples were fixed in 25% formalin for 24 h. After a dehydration process with ethanol at different concentrations and xylol, they were introduced into paraffin blocks at 65 °C, obtaining blocks from which 2 μm sections were made in an automatic microtome, which were stained with hematoxylin and eosin or immunostained with different antibodies, as described in section 7.5. The two pieces of the tumor destined for RNA and protein analysis were frozen in dry ice in cryotubes and were stored at −80 °C until pulverization with a mortar and liquid nitrogen.

### Immunohistochemistry

Samples were extracted from the different tissues of the mice and fixed in buffered 25% formalin for 24 h. After a dehydration process with ethanol at different concentrations and xylol, the samples were introduced in paraffin at 65 °C. We obtain 2 μm sections in an automatic microtome from each paraffin block. For staining with the antibodies KI67, CD45, F4/80, EMX2, and NES, the samples were rehydrated by the usual procedure and antigenic recovery was performed with citrate buffer at pH 6.5 (Dako/Agilent) heating in a pressure cooker for 2 min. In the case of the EMX1 antibody, antigenic recovery was carried out with EDTA buffer at pH 9 (Dako/Agilent). All primary antibodies were incubated overnight (12 h) in a humid chamber and at 4 °C, following the dilutions (Table 4). Then, the corresponding secondary antibody was added 1 h and a half at 1 : 400 in a humid chamber and at room temperature. The samples were washed with TBS + 0.1% Tween 20. After the endogenous peroxidase blocked with hydrogen peroxide and methanol (Dako/Agilent), 1% blocking solution was added (Sigma-Merck) and development was performed with the peroxidase system using diaminobenzidine plus (Dako/Agilent) as substrate. Counterstaining was performed with Harris hematoxylin. Finally, the sample was completely dehydrated with xylol and mounting was performed with Pertex. Photos were taken with an Olympus BX-61 microscope. The evaluation of the samples was carried out by quantitative microscopic analysis. Percentage values of immunostaining were assigned with respect to five fields.

### Analysis in public databases

The expression levels of EMX1/EMX2 and genes related to stem cell properties in public databases of human embryonic stem cells and human induced pluripotent stem cells were examined:

• Yamanaka, GEO ID: GSE9561.

• Thomson, GEO ID: GSE15148.

### Statistical analysis

We used the GraphPad Prism 6 software program for all statistical analyses of the experiments performed with the cell lines and the in vivo experiments. To analyze the differences in all the functional or transcriptomic tests carried out with the lines transfected with the cDNA of EMX1, EMX2, shRNAs, and the lines transfected with the different control vectors, we used Student’s *t*-test for unpaired samples or Student’s *t*-test with Welch’s correction. To determine the statistical significance of the survival graphs, we used a *χ*^2^-test (LogRank test). *P*-values < 0.05 were considered statistically significant and were represented according to the following classification: *p < 0.05, **p < 0.01, and ***p < 0.001.

## Supplementary information

SI guide

Supplemental material

## Data Availability

No datasets were generated during the current study. The datasets analyzed during the current study are publicly available in the different repositories.
