# Supplementary material for: Regulation of sarcomagenesis by the empty spiracles homeobox genes EMX1 and EMX2
Source: Cell Death Dis. 2021 May 20;12(6):515. doi: 10.1038/s41419-021-03801-w (PMC8137939; doi:10.1038/s41419-021-03801-w)
Supplement: Supplementary file 1 — SI guide [file 41419_2021_3801_MOESM1_ESM.pdf]

SUPPLEMENTARY INFORMATION

Supplementary Figure S1. Analysis of the expression levels and the methylation status of the promoter of EMX1 and EMX2 in the panel of sarcoma lines.

Supplementary Figure S2. Effect of EMX1 and EMX2 overexpression on the phenotype of clones in sarcoma cell models.

Supplementary Figure S3. Effect of the reduction of EMX1 and EMX2 on the phenotype of clones in sarcoma cell models.

Supplementary Figure S4. Immuno-phenotypic characterization of KI67 of the sarcomas obtained by the induction of 3MC in the KOs of Emx1 and Emx2.

Supplementary Figure S5. Immunophenotypic characterization of 3MC-induced sarcomas, in KO models.

Supplementary Figure S6. Analysis of the Prognosis value of EMX1/EMX2 genes in different public datasets.
