# Supplementary material for: Regulation of sarcomagenesis by the empty spiracles homeobox genes EMX1 and EMX2
Source: Cell Death Dis. 2021 May 20;12(6):515. doi: 10.1038/s41419-021-03801-w (PMC8137939; doi:10.1038/s41419-021-03801-w)
Supplement: Supplementary file 2 — Supplemental material [file 41419_2021_3801_MOESM2_ESM.doc]

**SUPPLEMENTARY INFORMATION**

**
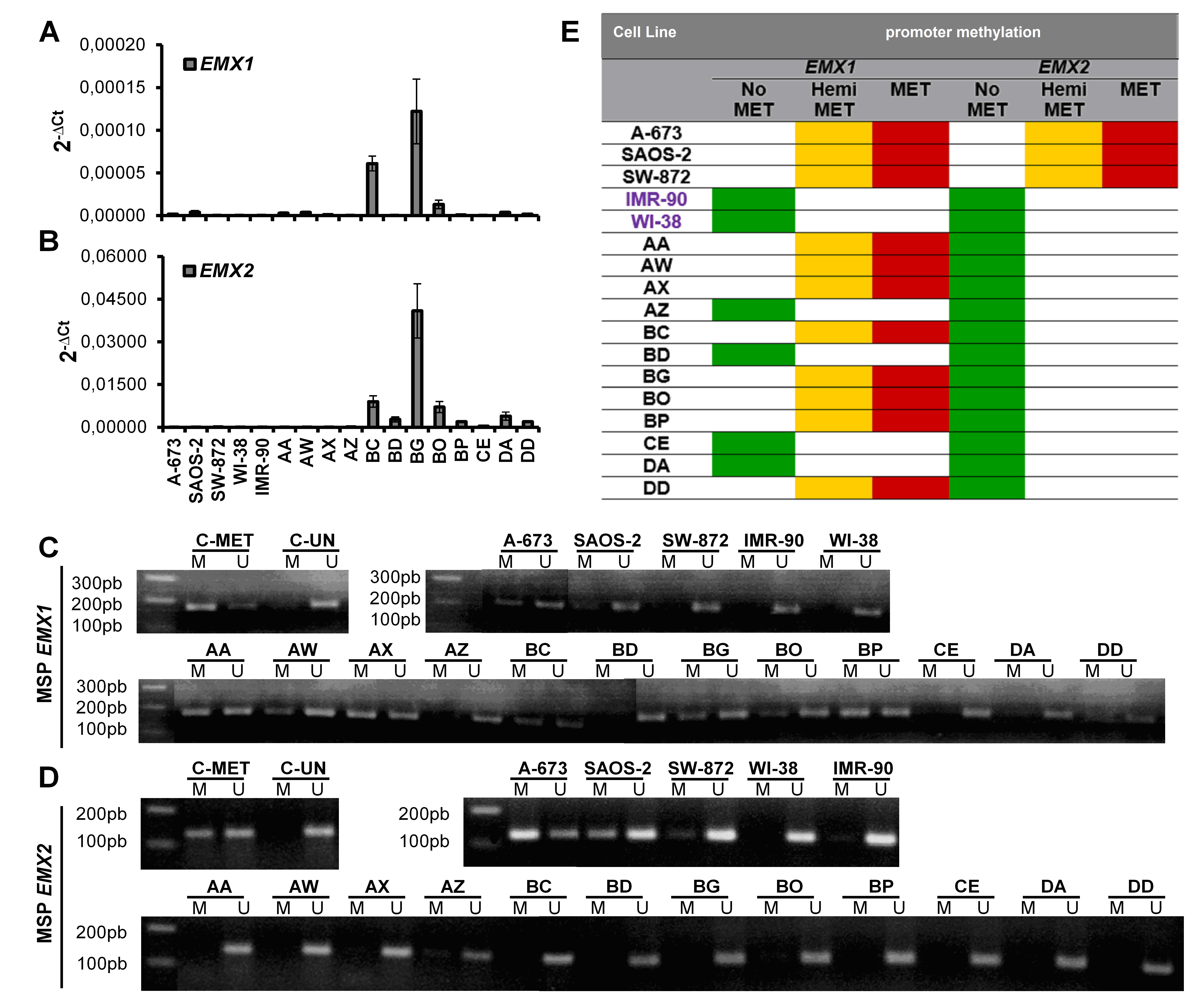
**

**Supplementary Figure S1. Analysis of the expression levels and the methylation status of the promoter of EMX1 and EMX2 in the panel of sarcoma lines.** (A and B) Graphs of the expression levels (2-ΔCt) of EMX1 and EMX2 , measured by qRT-PCR. The mean of 3 independent experiments is represented, in triplicate ± standard deviation. The description of the cell lines is presented in the Table 3 of the manuscript.

**
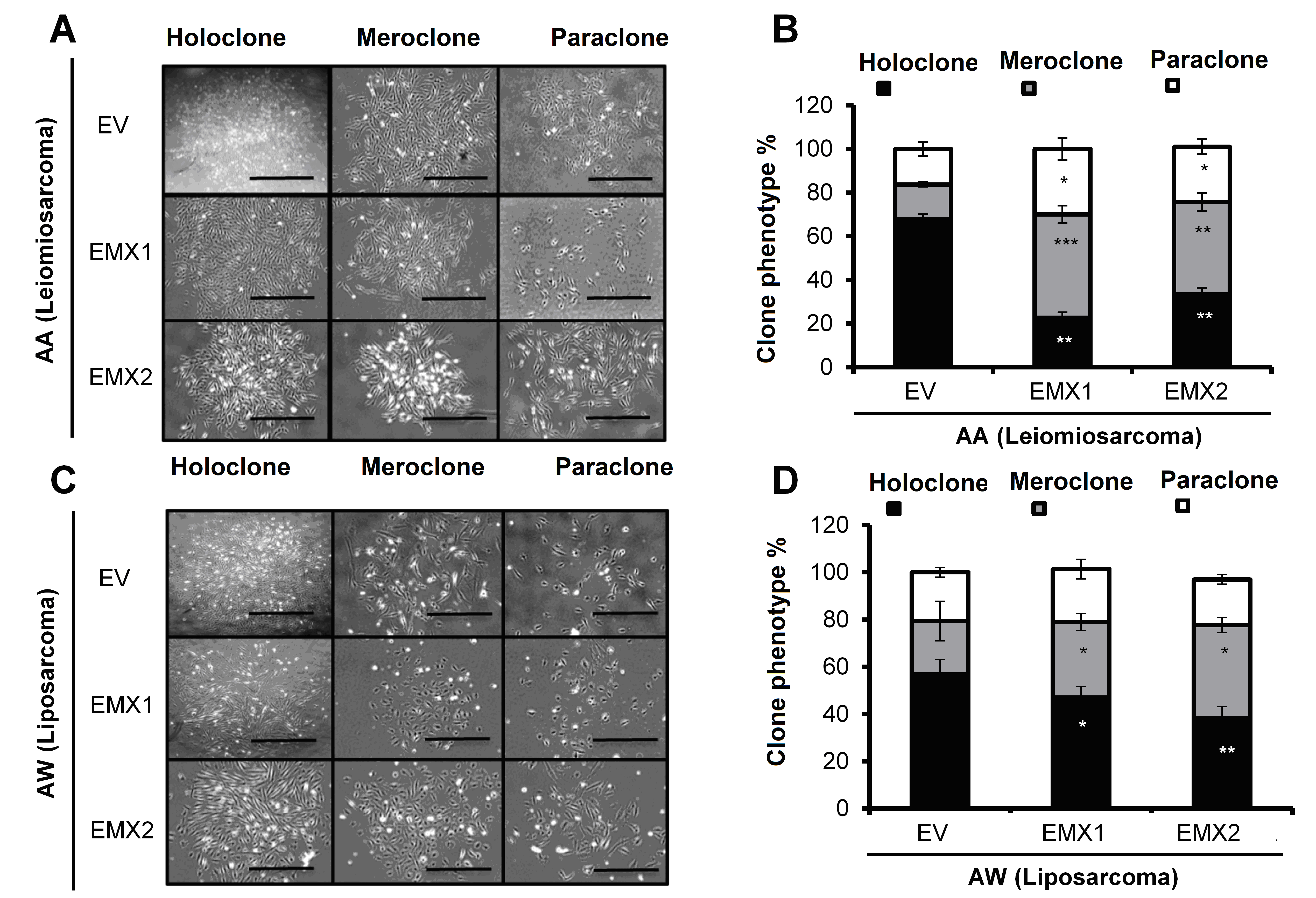
**

**Supplementary Figure S2. Effect of EMX1 and EMX2 overexpression on the phenotype of clones in sarcoma cell models**. Results of the analysis of clonal phenotypes in the overexpression models of the sarcoma line AA (upper panel) and AW (lower panel). In (A) and (C) the different clonal phenotypes are indicated according to the properties of the stem cells and the degree of differentiation: holoclone, meroclone and paraclone. The bar corresponds to 200µm. In (B) and (D) the percentage of clonal types. The mean of 3 independent experiments is represented, in triplicate ± standard deviation. Statistical analysis was performed with the Student's t test (* p <0.05; ** p <0.01; *** p <0.001).


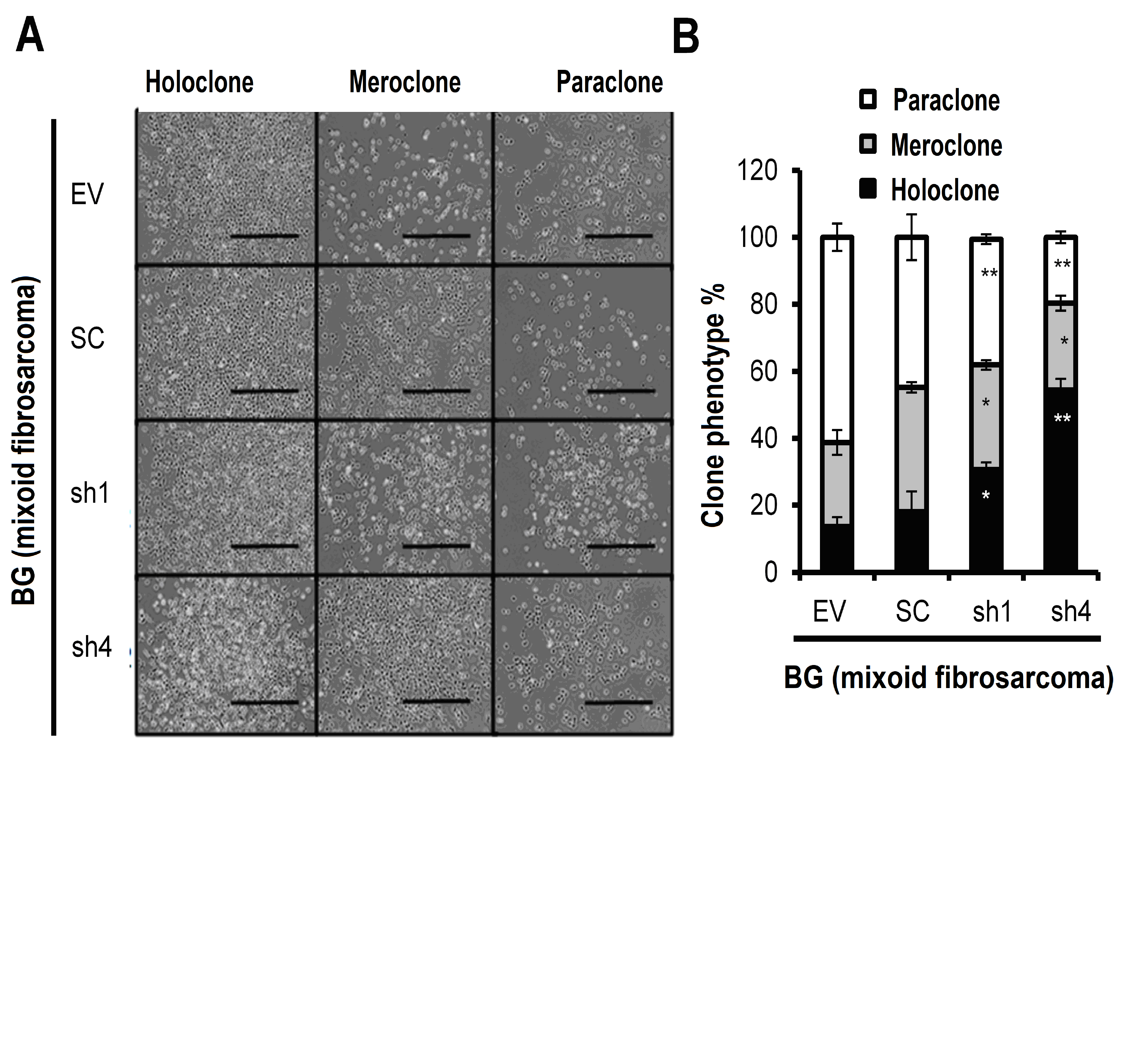


**Supplementary Figure S3. Effect of the reduction of EMX1 and EMX2 on the phenotype of clones in sarcoma cell models**. (B) Clonability assay in the EMX1 / EMX2 silencing model of the BG sarcoma line. In (B) the number of colonies is indicated. In (C) the graph of the size of the colonies is indicated. (A) Attached are some images of the clonability assay plates stained with crystal violet on the last day of the experiment. In (D) the different clonal phenotypes are indicated according to the stem cell properties and the degree of differentiation: holoclone, meroclone and paraclone. (E) Graph of the percentage of clonal types. The bar corresponds to 200µm. The mean of 3 independent experiments is represented, in triplicate ± standard deviation. Statistical analysis was performed with the Student's t test (* p <0.05; ** p <0.01; *** p <0.001).


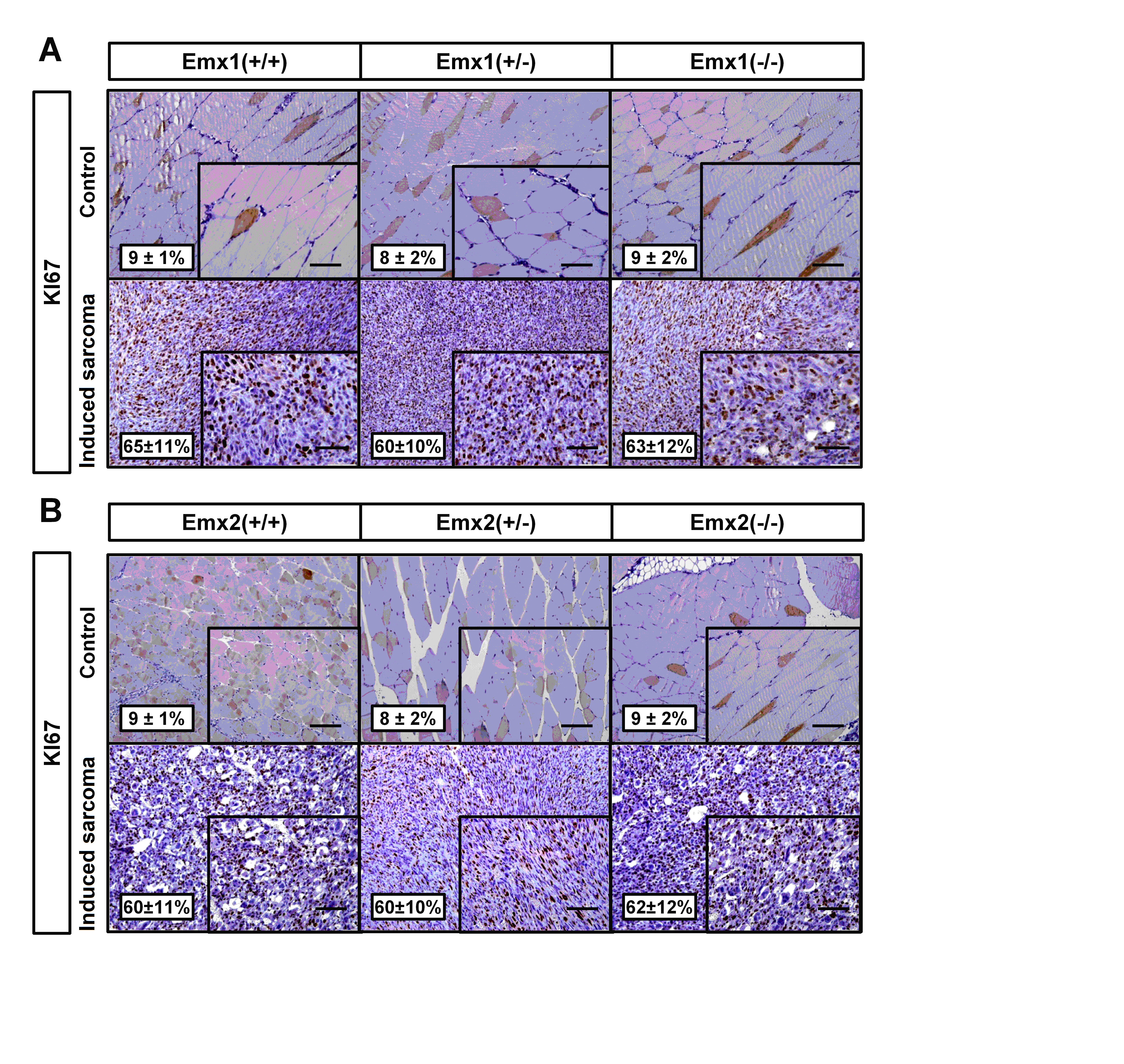


**Supplementary Figure S4. Immuno-phenotypic characterization of KI67 of the sarcomas obtained by the induction of 3MC in the KOs of Emx1 and Emx2.** (A and B) Result of immunohistochemistry against the cell proliferation marker KI67. The upper panel shows micrographs of the section of the control muscle tissue at 200x magnification and the lower panel those of the sarcoma induced by 3MC, in the murine models of Emx1 (A) and Emx2 (B). The image in the lower right corner of each micrograph is 400x magnification. The bar indicates 200 µm. The percentage of mean cell immunolabeling measured in 5 fields per replicate ± the standard deviation is monitored. Immunohistochemistry was performed in a minimum of 3 replications for each sample.


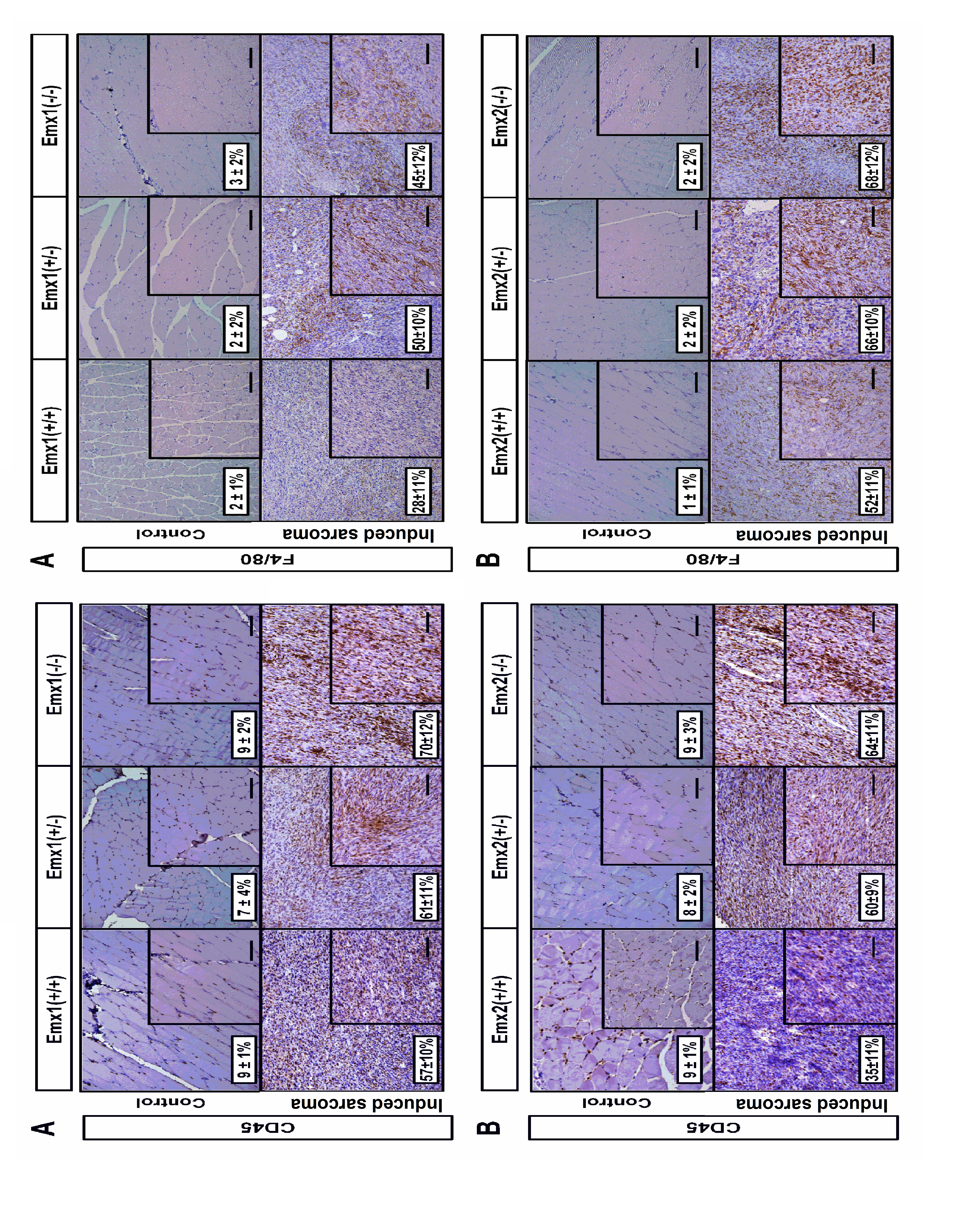


**Supplementary Figure S5. Immunophenotypic characterization of 3MC-induced sarcomas, in KO models.** (A and B) Result of immunohistochemistry against the CD45 cell proliferation marker. The upper panel shows micrographs of the section of the control muscle tissue at 200x magnification and the lower panel those of the sarcoma induced by 3MC, in the murine models of Emx1 (A) and Emx2 (B). The image in the lower right corner of each micrograph is 400x magnification. The bar indicates 200 µm. The percentage of mean cell immunolabeling measured in 5 fields per replicate ± the standard deviation is monitored. Immunohistochemistry was performed in a minimum of 3 replications for each sample.
Immunophenotypic characterization of F4 / 80 of sarcomas induced by 3MC, in KO models. (A and B) Result of immunohistochemistry against the cell proliferation marker F4 / 80. The upper panel shows micrographs of the section of the control muscle tissue at 200x magnification and the lower panel those of the sarcoma induced by 3MC, in the murine models of Emx1 (A) and Emx2 (B). The image in the lower right corner of each micrograph is 400x magnification. The bar indicates 200 µm. The percentage of mean cell immunolabeling measured in 5 fields per replicate ± the standard deviation is monitored. Immunohistochemistry was performed in a minimum of 3 replications for each sample.


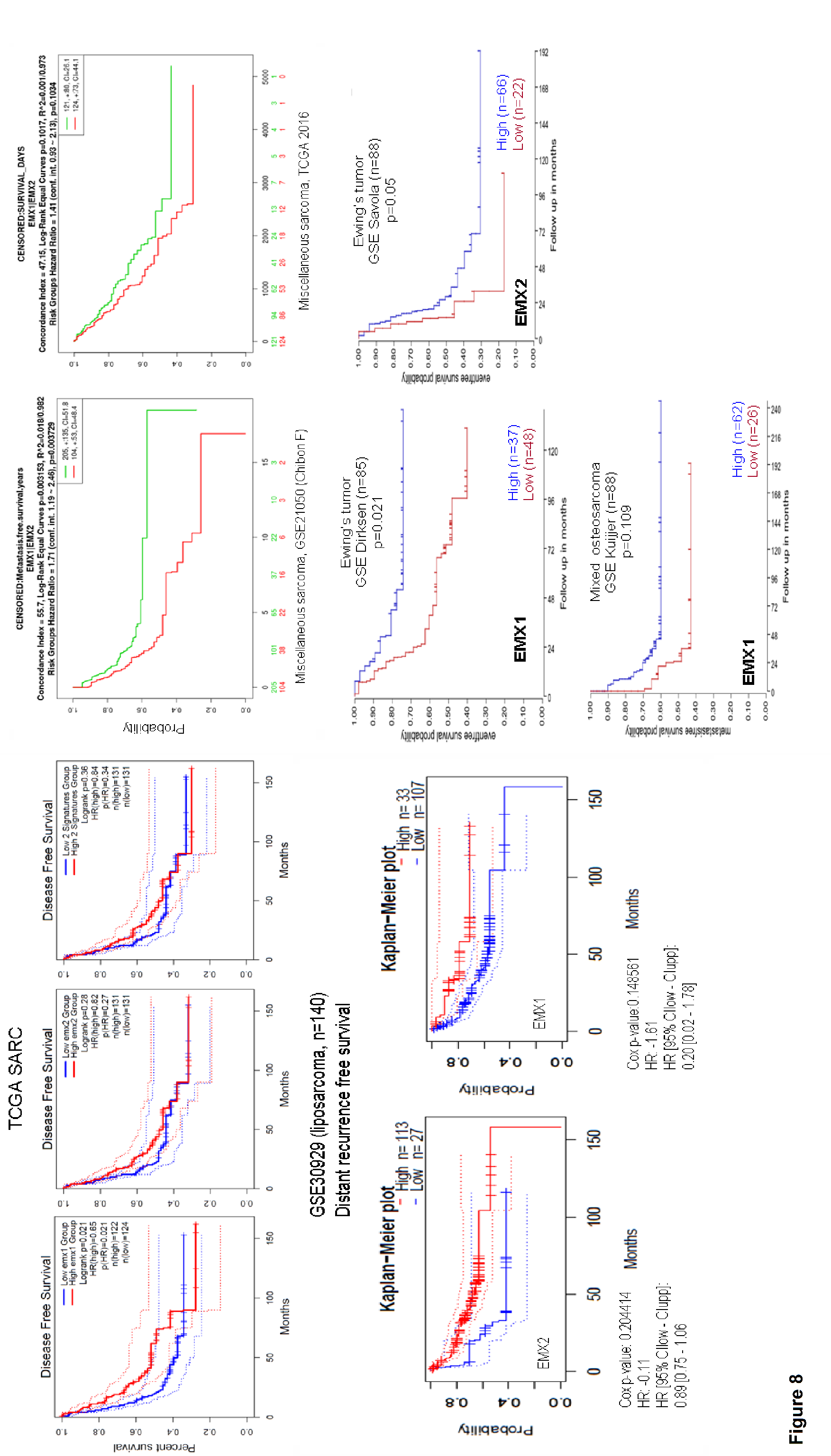


**Figure supplementary FS6:** Analysis of the Prognosis value of EMX1/EMX2 genes in different public datasets (included in the figure). Kaplan Meier curves are shown stratifying patients for levels of EMX mRNA expression as high, (red) or low (high) from the median levels.

To explore the relevance of the expression of EMX1 and EMX2, we measured its expression on different public database and correlated this with the probability of survival of the patients. Overall survival corresponds to the time elapsed between the date of diagnosis or surgery and the last date of follow-up of the patients. It was analyzed in different database platforms: those for Ewing's sarcoma, osteosarcoma, and mixed sarcoma in TCGA datasets, analyzed in different platforms, the R2-Genomics platform, SurvExpress and GEPIA2. In general terms, a reduction in disease free survival or/and overall survival was observed in the group of patients with low levels of EMX1 and EMX2. These differences are significant or close to statistical significance, with a clear tendency and stratification of patients.
